# Characterization of the Ubiquitin C-Terminal Hydrolase and Ubiquitin-Specific Protease Families in Rice (*Oryza sativa*)

**DOI:** 10.3389/fpls.2018.01636

**Published:** 2018-11-15

**Authors:** Dong-Hui Wang, Wei Song, Shao-Wei Wei, Ya-Feng Zheng, Zhi-Shan Chen, Jing-Dan Han, Hong-Tao Zhang, Jing-Chu Luo, Yong-Mei Qin, Zhi-Hong Xu, Shu-Nong Bai

**Affiliations:** ^1^State Key Laboratory of Protein and Plant Gene Research, College of Life Science, Peking University, Beijing, China; ^2^National Center of Plant Gene Research, Beijing, China; ^3^State Key Laboratory of Systematic and Evolutionary Botany, Institute of Botany, Chinese Academy of Sciences, Beijing, China

**Keywords:** ubiquitin C-terminal hydrolase, ubiquitin-specific protease, enzyme activity, rice, stamen, male fertility

## Abstract

The ubiquitin C-terminal hydrolase (UCH) and ubiquitin-specific processing protease (UBP) protein families both function in protein deubiquitination, playing important roles in a wide range of biological processes in animals, fungi, and plants. Little is known about the functions of these proteins in rice (*Oryza sativa*), and the numbers of genes reported for these families have not been consistent between different rice database resources. To further explore their functions, it is necessary to first clarify the basic molecular and biochemical nature of these two gene families. Using a database similarity search, we clarified the numbers of genes in these two families in the rice genome, examined the enzyme activities of their corresponding proteins, and characterized the expression patterns of all *OsUCH* and representative *OsUBP* genes. Five *OsUCH* and 44 *OsUBP* genes were identified in the rice genome, with four OsUCH proteins and 10 of 16 tested representative OsUBP proteins showing enzymatic activities. Two *OsUCH*s and five *OsUBP*s were found to be preferentially expressed in the early development of rice stamens. This work thus lays down a reliable bioinformatic foundation for future investigations of genes in these two families, particularly for exploring their potential roles in rice stamen development.

## Introduction

Understanding transcriptional regulation is important for elucidating how genetic information determines phenotypes; however, numerous regulatory layers other than transcription play roles in the flow of information from genes to phenotypes, which interact to maintain or enhance both robustness and adaptability. Proteins execute diverse biological functions; therefore, protein stability is a critical checkpoint regulating this flow of information, and protein ubiquitination and deubiquitination have been intensively investigated (Zhang, 2003; Frappier and Verrijzer, 2011).

Ubiquitination plays a key role in protein degradation (Ciechanover, 1998; Weissman, 2001), while deubiquitination has the opposite role, with deubiquitinating enzymes (DUBs) cleaving ubiquitin from ubiquitin-conjugated proteins (Chung and Baek, 1999; Wilkinson, 2000). Rawlings and Barrett (1993) performed a systematic analysis of over 600 peptidases available at the time, classifying them into 84 distinct families. The DUBs were classified as C-type (cysteine) peptidases. Based on their catalytic mechanisms and amino acid sequences, Amerik and Hochstrasser (2004) classified the DUBs into five subfamilies, including four special types of cysteine proteases; ubiquitin C-terminal hydrolase (UCH), ubiquitin-specific processing protease (UBP or USP), otubain protease (OUT), and Machado-Joseph disease protease (MJD), as well as one zinc-dependent metalloprotease, JAB1/MPN/Mov34 metalloenzyme (JAMM). Recently, two new DUB subfamilies, monocyte chemotactic protein-induced protein (MCPIP) and the motif interacting with Ub-containing novel DUB family (MINDY), were identified, expanding the DUBs into seven subfamilies (Liang et al., 2010; Fraile et al., 2012; Abdul et al., 2016). The largest and most diverse subfamily among them is the UBP group. Cysteine proteases are classified based on the characteristic amino acids at their active sites: UCH has the amino acids Q84, C90, H161, and D176, annotated as the C12 domain, while UBP is characterized by the amino acids N109, C114, H435, and D/N451, known as the C19 domain (MEROPS database^1^).

Protein deubiquitination is much less well-understood than protein ubiquitination and degradation; however, some DUBs are known to be involved in a wide range of biological processes in animals, fungi, and plants (Nijman et al., 2005; Reyes-Turcu et al., 2009; Kouranti et al., 2010). These biological processes include transcriptional regulation and DNA repair (Nijman et al., 2005; Huang et al., 2006; Clague et al., 2012), chromatin modification (Sridhar et al., 2007; Reyes-Turcu et al., 2009), protein degradation (Yan et al., 2000; Guterman and Glickman, 2004; Wu et al., 2004), signal transduction (Moon et al., 2005; Reyes-Turcu et al., 2009; Ewan et al., 2011; Clague et al., 2012; Cui et al., 2013), animal cell growth, apoptosis, neural and germ cell differentiation (Nijman et al., 2005; Kim et al., 2007; Zhang et al., 2007; Bedard et al., 2011; Hou and Yang, 2013), and plant morphogenesis and programmed cell death (Doelling et al., 2001; Yang et al., 2007; Liu et al., 2008; Ewan et al., 2011; Ramakrishna et al., 2011; Cui et al., 2013; Du et al., 2014). These diverse functions highlight the importance of protein deubiquitination in biological processes.

Previously, 21 genes encoding UBP proteins were reported in the rice (*Oryza sativa*) genome (Moon et al., 2009). In our previous rice stamen gene expression profiles (Lu et al., 2006; Chen et al., 2015), we identified a gene, Os11g38610 (AK101994), specifically expressed in rice stamens that was annotated as *OsUCH*. Considering the reported functions of UCH in gonadal transformation and spermatogenesis in animals (Kwon et al., 2004; Wang et al., 2006; Sun et al., 2008; Luo et al., 2009), such a finding led us to further investigate whether the UCH genes play a role in the regulation of stamen development in rice.

It was surprise, however, that detail sequence analysis revealed that neither C12, nor C19 hallmark sequence of *OsUCH* and *OsUB*P was found in the sequence of Os11g38610 ^2^. When we investigated how many genes encoding DUBs were present in the rice genome, the three major databases (NCBI, RIKEN, and MSU) gave different outputs, indicating that more reliable information about the classification of DUBs is required for the functional and mechanistic investigation of these genes in rice. Here, we determine that there are five *OsUCH* and 44 *OsUBP* genes in the rice genome, based on their characteristic C12 and C19 domains, respectively. The 44 OsUBP proteins could be classified into 14 groups according to their sequence similarity. Four of the five OsUCH and 10 of 16 selected OsUBP proteins had DUB enzyme activity, and one, OsUCH3, was found to have a stronger enzymatic activity than its homologs in yeast (*Saccharomyces cerevisiae*) and humans (*Homo sapiens*). In addition, among these genes, some were found to be preferentially expressed during stamen development. This work thus lays down a reliable bioinformatic foundation for future investigations of genes in these two families, particularly for exploring their potential roles in rice stamen development.

## Materials and Methods

### Plant Materials

The rice (*Oryza sativa* L. ssp. *japonica* cv. *Zhonghua11*) cultivars were provided by the Chinese Academy of Agricultural Sciences. The rice seeds were soaked in water at 28°C for about 2 weeks, then the seedlings were transplanted into plastic pots and cultured at 28 ± 2°C under an 11-h light/13-h dark photoperiod in a greenhouse, or transplanted into the experimental field during the normal growing season in Beijing, China. The tissues for the expression analyses were harvested and immediately frozen in liquid nitrogen, then stored at −80°C until required.

### Phylogenetic Analysis

For the phylogenetic analysis of the OsUCH/OsUBP protein family members, we performed a database similarity search using the C12 and C19 domains as query sequences against the UniProt database (The UniProt Consortium, 2012). Full-length amino acid sequences for *Arabidopsis thaliana, Saccharomyces cerevisiae, Schizosaccharomyces pombe, Physcomitrella patens, Selaginella moellendorffii, Zea mays, Oryza sativa (osm), Sorghum bicolor, Populus trichocarpa, Monopterus albus, Mus musculus (mou), Rattus norregicus*, and *Homo sapiens* were retrieved from the NCBI website^3^ and UniProt database (The UniProt Consortium, 2012). The phylogenetic analyses were conducted using the RAxML software (Stamatakis, 2006; Ott et al., 2007), based on the maximum likelihood method with the WAG model, followed by rapid bootstrapping tests.

### cDNA Preparation and RT-qPCR Analysis

Total RNA was extracted with the RNeasy Plant Mini Kit (Qiagen), according to the manufacturer’s protocol. cDNA was obtained by reverse transcribing 0.5–1.0 mg RNA with SuperScript III Reverse Transcriptase (Life Technologies).

Tissue samples, including five stamens at developmental stages 2–6 (S2–S6), leaves, leaf primordia, SAMs, and RAMs, were extracted as described by Chen et al. (2015). The cDNA templates, primers, and SYBR Premix Ex Taq (Takara) were mixed and a quantitative PCR was performed using an Applied Biosystems 7500 Real-Time PCR System with three technical replicates. The data were processed using the 7500 software (ver. 2.0) based on the ddCt method, normalizing the expression data to that of the reference gene *GAPDH*, which was determined using the primers developed by Ji et al. (2014). The primers used for RT-qPCR analysis are listed in Supplementary Table S9.

### *In situ* Hybridization

The *in situ* hybridization was performed according to Bai et al. (2004), with some modifications. For the preparation of the paraffin sections, the inflorescences were infiltrated under vacuum in FAA (4% w/v), 3.7% paraformaldehyde, and 0.25% glutaraldehyde in 0.1 M sodium phosphate buffer (pH 7.4), then incubated overnight at 4°C. The samples were then dehydrated through a series of graded ethanol concentrations and a xylene series, before being embedded in Paraplast Plus (Sigma) and sliced into 7.5-μm sections. Digoxygenin-labeled antisense RNA probes were generated by *in vitro* transcription, according to the instructions provided with the DIG RNA Labeling Kit (SP6/T7; Roche). The samples were deparaffinized by rinsing in xylene and then dehydrated through a graded ethanol series. For the hybridization, the sections were incubated at 45°C overnight with hybridization buffer [500 ng ml^−1^ digoxygenin-labeled RNA, 50% formamide, 300 mM NaCl, 1 mM EDTA, 1x Denharts, 10% dextransulphate, 10 mM DTT, 250 ng ml^−1^ tRNA, and 100 μg ml^−1^ Poly(A)] and covered with a slide. After hybridization, the cover slide was removed in 2x SSC at room temperature and the sections were washed twice for 30 min at 45°C with maleic buffer (100 mM maleic acid and 150 mM NaCl, pH 7.5), then treated with RNase A (20 μg ml^−1^ in 500 mM NaCl/TE, pH 7.5) at 37°C for 30 min, before being washed twice in 500 mM NaCl/TE (pH 7.5) at room temperature. The hybridized probes were detected using an anti-digoxigenin-Ap antibody and then visualized with 4-nitroblue tetrazolium chloride and 5-bromo-4-chloro-3-indolyl-phosphate (NBT/BCIP), according to the protocol developed by Roche. Photos were taken under Imager D2 (Carl Zeiss). The primers used for *in situ* hybridization are listed in Supplementary Table S9.

### Protein Purification

The OsUCH and OsUBP proteins were expressed by the pCold TF cold shock expression system (TAKARA) in *Escherichia coli*. *OsUCH3* was cloned into the pCold TF vector then transformed into *Trans*B (DE3) chemically competent cells (TransGen). The pCold empty vector, which expressed only the trigger factor and the 6^∗^his-tag, was used as the negative control. The cells were induced to express the transgenic material by adding IPTG at a final concentration of 0.5 mM, followed by an incubated at 15°C for 24 h.

Cells were collected by centrifugation and sonicated in a lysis buffer containing 20 mM NaH_2_PO_4_, 500 mM NaCl, and 20 mM imidazole (pH 7.4). The cell extract was purified using His Trap HP (GE HEALTHCARE), following the manufacturer’s instructions. Protein concentrations were determined using the Bradford Protein Assay Kit (TransGen) and diluted to 50 μg ml^−1^.

### *In vitro* Assay of OsUCH Hydrolytic Activity

The *in vitro* assay of OsUCHs hydrolytic activity were performed according to the previously published protocols (Dang et al., 1998; Artavanis-Tsakonas et al., 2010), with some modifications. To test the DUB activity and the deneddylating activity of the OsUCHs *in vitro*, 2 μl 50 μg ml^−1^ OsUCH3 recombinant protein was added to 2 ml reaction buffer containing 50 mM Tris-HCl, 0.5 mM EDTA, and 5 mM DTT (pH 8.0). The reaction was initiated by adding 2 μl of the corresponding substrate, Ub-AMC or NEDD8-AMC (BostonBiochem), at 10 μM and monitored using an RF-5301PC fluorescence spectrophotometer (Hitachi). The free AMC was excited at 345 nm and its fluorescence emission rate was recorded at 445 nm.

The trigger factor with the 6^∗^his-tag, which was expressed by the pCold TF empty vector, was used as a negative control, while 0.5 μg ml^−1^ recombinant HsUCHL3 (BostonBiochem) was used as a positive control.

### *In vivo* Assay of Hydrolytic Activity

The *in vivo* assay of OsUCHs and OsUBPs hydrolytic activity were performed according to the previously published protocols (Yan et al., 2000; Yang et al., 2007; Liu et al., 2008), with some modifications. To test the DUB activity of the OsUCHs or OsUBPs *in vivo*, the *OsUCH* or *OsUBP* sequences were co-expressed with the substrate *AtUBQ10* in pETDuet-1 which has the advantage of having two polyclonal sites (MCS), and can express two proteins at the same time in *Trans*B (DE3). The six ubiquitin gene UBQ10 was inserted into MCS1 and the UCH or UBP gene into MCS2. The pETDuet-1 empty vector and the pETDuet-1 vector containing only *AtUBQ10* were used as the negative controls. The cells were induced by adding IPTG at a final concentration of 0.5 mM, followed by a 10 h incubation at 28°C. The cell culture centrifuge and add 5x SDS-PAGE loading buffer, then subjected to SDS-PAGE and transferred to a PVDF membrane (Millipore), detected using anti-ubiquitin antibodies (SantaCruz). Western blotting was carried out as described by Gu et al. (2011) with modifications: primary antibody in a 1:1000 dilution (SantaCruz) and the secondary antibody (Promega) was added at concentration of 1:2000. The rice OsUBP6 (Os01g36930) protein was a positive control (Moon et al., 2009).

### Accession Numbers

All of the gene accession numbers presented in the text are available in the Supplemental Information (Supplementary Tables S1–S5). They are derived from the MSU Rice Genome Annotation Project (MSU 6.1), Rice Annotation Project (RAP, IRGSP 1.0), or NCBI GenBank. Sequence data from this article can be found in the Rice Genome Annotation Project data libraries.

## Results and Discussion

### The Rice Genome Contains 5 *OsUCH* and 44 *OsUB*P Genes

To determine how many *OsUCH* and *OsUBP* genes are present in the rice genome, we performed a keyword search against the NCBI protein database^3^ using “Ubiquitin carboxyl-terminal hydrolase” and “Ubiquitin-specific-processing protease” as queries. A total of 135 sequences were retrieved, 35 of which were identified as genes encoding OsUCH and/or OsUBP proteins (Supplementary Table S1). A similar result was obtained from the rice specific database Oryzabase^4^. However, a search of the Gramene^5^ database retrieved 19 *OsUCH* and 40 *OsUBP* genes, including Os11g38610 (Table 1). The criteria proposed by Rawlings and Barrett (1993) from their evolutionary analysis of peptidases were adapted by the MEROPS database^6^; therefore, we performed a similar search for rice *OsUCH* and *OsUBP* genes in this database, identifying six genes annotated as *OsUCH* and 22 classified as *OsUBP* (Table 1). Clearly, a simple database search using “Ubiquitin carboxyl-terminal hydrolase” and “Ubiquitin-specific-processing protease” as queries could not yield consistent results for the determination of the numbers of *OsUCH*- and *OsUBP*-family members in rice.

**Table 1:** The number of rice *OsUCH* and *OsUBP* genes present in different databases.

Tool or Database	*OsUCH*s	*OsUBP*s	Total
MSU Rice	19	40	59
MEROPS	6	22	28
InterProScan	5	44	49
Pfam	5	49	54

To solve this problem, we performed a sequence similarity search against the Pfam and InterPro datasets, obtained from the UniProt database (The UniProt Consortium, 2012), using the C12 and C19 domains as query sequences (Zdobnov and Apweiler, 2001; Punta et al., 2012). In this search, we identified five proteins containing the C12 domain, indicating that there are five *OsUCH* genes in the rice genome (Table 1). A total of 49 proteins containing the C19 domain were found in Pfam, while 44 were identified in the InterPro dataset (Table 1). We took a more stringent criterion and propose that there are 44 *OsUBP* genes in the rice genome (Supplementary Table S2). This dataset contains all *OsUBP* genes reported by Moon et al. (2009), except for Os08g41640 which contains neither a C12 nor a C19 domain.

### Phylogenetic Relationships Among the Members of the *OsUCH* and *OsUBP* Families

To analyze the relationships between the rice genes encoding the OsUCH and OsUBP proteins, we performed two phylogenetic analyses on 60 UCH protein sequences and 202 UBP protein sequences obtained from 13 and 12 species, respectively, of fungi, animals, and plants, retrieved from the UniProt database (Supplementary Table S3).

**FIGURE 1:**
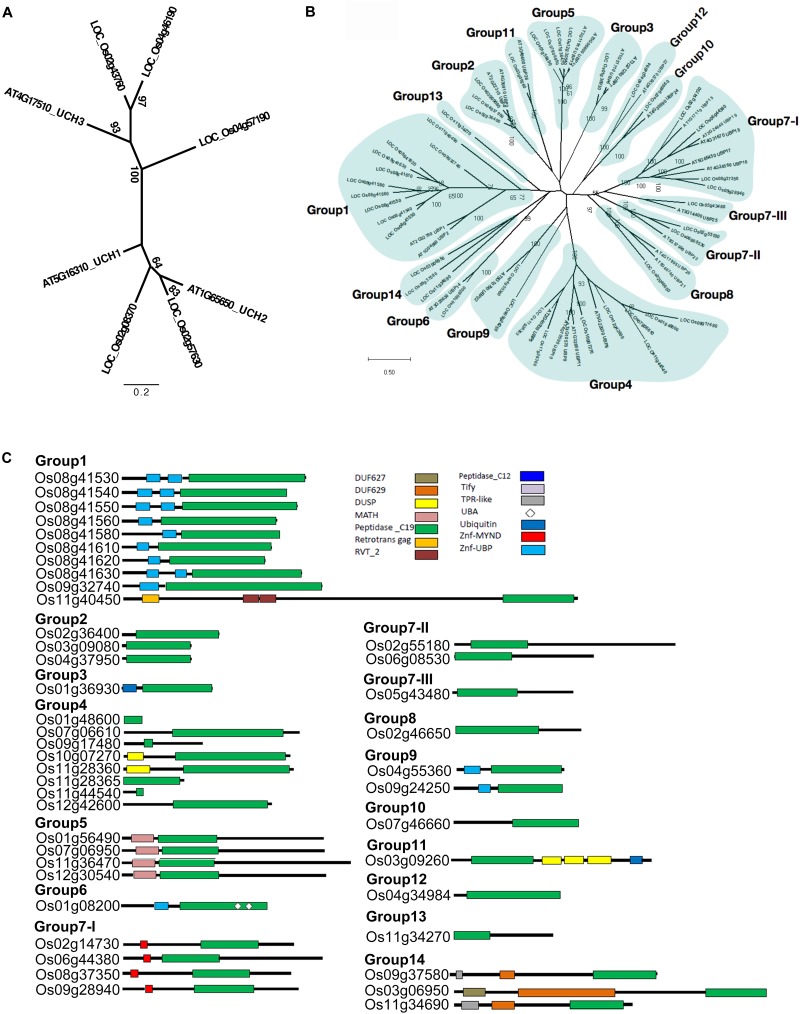
Phylogenetic relationships and domain architectures of UCH and UBP protein family in Arabidopsis and rice. **(A)** ML unrooted tree of UCH protein family. **(B)** ML unrooted tree of UBP protein family. **(C)** Based on the predicted amino acid sequence identities and domain structures, the 44 rice OsUBP genes can be subdivided into 14 groups (G1–G14). The domains are shown in different colored boxes. The G1 members (Os08g41530, Os08g41540, Os08g41550, Os08g41560, Os08g41580, Os08g41610, Os08g41620, Os08g41630, Os09g32740, and Os11g40450) contain one peptidase_C19 domain or up to two zinc-finger ubiquitin-specific protease (ZnF-UBP) domains. The three G2 members (Os03g09080, Os04g37950, and Os02g36400) contain the peptidase_C19 domain. The G3 member (Os01g36930) contains a single peptidase_C12 and a single peptidase_C19 domain. The G4 members (Os10g07270, Os11g28360, Os12g42600, Os11g44540, Os01g48600, Os07g06610, Os09g17480, and Os11g28365) have a peptidase_C19 domain and up to one DUSP (domain in USPs) motif. The G5 OsUBPs (Os01g56490, Os12g30540, Os11g36470, and Os07g06950) have a single meprin and TRAF homology (MATH) domain in their N-terminal(regions and a peptidase_C19 domain. The G6 OsUBP (Os01g08200) members contain ZnF-UBP, a peptidase_C19 domain, and two ubiquitin-associated (UBA) domains. The G7-I (Os09g28940, Os08g37350, Os06g44380, and Os02g14730) proteins contain a myeloid, nervy and DEAF1 (MYND)-type zinc finger (ZnF-MYND) motif and a peptidase_C19 domain, and the G7-II (Os02g55180 and Os06g08530), G7-III (Os05g43480), and G8 (Os02g46650) proteins have a peptidase_C19 domain in their N-terminal regions. The G9 (Os04g55360 and Os09g24250) proteins contains ZnF-UBP and a peptidase_C19 domain, while the G10 member (Os07g46660) contains a peptidase_C19 domain in its C-terminal region. The G11 (Os03g09260) protein contains a peptidase_C19 domain, three DUSP (domain in USPs) motifs, and a ubiquitin motif. The G12 member (Os04g34984) contains a peptidase_C19 domain, while the G13 protein (Os11g34270) contains a peptidase_C19 domain and a ZnF-UBP domain. The G14 members (Os09g37580, Os03g06950, and Os11g34690) contain TPR-like (or Tify), DUF629 and peptidase_C19 domains. DUF627, DUF629, MATH, peptidase_C19, retrotrans gag, RVT_2, peptidase_C12, Tify, TPR-like, UBA, Ubiquitin, ZnF-MYND, and ZnF-UBP domains are indicated in the upper part of the figures.)

According to our phylogenetic analysis, the 60 genes annotated as UCH were clustered into two clades (Supplementary Figure S1). Two rice *OsUCH* genes (Os02g08370 and Os02g57630) were grouped in the clade with Arabidopsis (*Arabidopsis thaliana*) *AtUCH1* (At5g16310) and *AtUCH2* (At1G65650), while the other three (Os02g43760, Os04g46190, Os04g57190) fell into the same clade as the genes involved in animal gonadal development and spermatogenesis, such as mouse (*Mus musculus*) *UCHL1* and *UCHL3* (Kwon et al., 2004; Wang et al., 2006; Luo et al., 2009). Following the Arabidopsis nomenclature, Os02g08370 and Os02g57630 were designated as *OsUCH1* and *OsUCH2*, respectively, while Os02g43760, Os04g46190, and Os04g57190 were designated as *OsUCH3, OsUCH4*, and *OsUCH5*, respectively (Figure 1A).

The phylogenetic tree was highly complicated for the OsUBPs (Supplementary Figure S2). To facilitate a simple comparison, we used the 27 Arabidopsis *UBP* genes as a reference and classified the rice *OsUBP* genes into 14 groups ((Figures 1B,C and Supplementary Table S4). Such a classification can be used to select representative samples for biochemical analysis.

In addition, we identified 39 genes coding for OTU, 16 for JAMM, but none for MJD or MCPIP in the rice genome (Supplementary Table S5).

### Enzyme Activity Was Detectable in Four of the Five OsUCH Proteins

**FIGURE 2:**
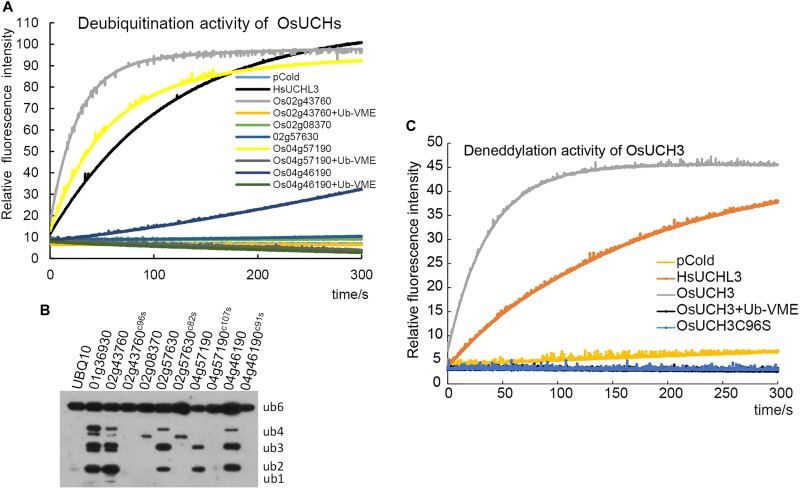
Enzyme activity assays of all OsUCHs. **(A)** Four OsUCHs show deubiquitination activity *in vitro*, using Ub-AMC as the substrate. When the DUB inhibitor Ub-VME was added, the OsUCHs completely lost their enzymatic activity. HsUCHL3 was used as a positive control. **(B)** Four OsUCHs show DUB activity *in vivo*, detected by the degradation of the substrate AtUBQ10 into small fragments. The active-site mutant protein OsUCH3^C96S^ had no enzymatic activity. OsUBP6 (Os01g36930) was used as a positive control. **(C)** OsUCH3 possesses deneddylation activity *in vitro*, using NEDD8-AMC as the substrate. When the DUB inhibitor Ub-VME was added, OsUCH3 completely lost its enzymatic activity. The active-site mutant protein OsUCH3^C96S^ had no enzymatic activity. HsUCHL3 was used as the positive control.

We next examined the enzyme activity of all five OsUCHs. Two methods have been widely used for assaying the enzyme activity of the DUBs; either the enzyme activities of the recombinant protein are directly measured using fluorescence spectrometry (Dang et al., 1998; Nishikawa et al., 2003), or the enzyme activity is assessed in the cell lysate of *Escherichia coli* transformed with *OsUCH* or *OsUB*P genes (Yan et al., 2000). Using both of these *in vitro* and *in vivo* assays, we demonstrated that the presence of deubiquitination activity in four of the five OsUCHs (Figures 2A,B); however, such enzymatic activity was not detected for Os02g08370 (AK067359).

We selected OsUCH3 for a more in-depth analysis of the biochemical characteristics of the rice OsUCH proteins, because it is preferentially expressed in the stamen and because its animal homologs are involved in spermatogenesis. Computational modeling showed little difference between the predicted 3D structure of OsUCH3 and that of its homolog proteins in other species (Supplementary Figure S3). The enzymatic activity of other UCH proteins is dependent on a cysteine in the catalytic triad of the protein (Erez et al., 2009; Supplementary Figure S4). To verify the conservation of the mechanism of enzymatic activity in OsUCH3, we generated a point mutation at the corresponding C96 residue (Supplementary Figure S5), replacing the cysteine with serine (C to S; OsUCH3^C96S^). Although no significant structural changes were observed (Supplementary Figure S6), the enzyme activity was complete lost in OsUCH3^C96S^ (Figures 2A,C). This suggests that C96 is a vital amino acid for OsUCH3 enzymatic activity, as has been reported for other proteins with similar sequences (Yan et al., 2000; Yang et al., 2007; Liu et al., 2008).

Using a standard assay (Dang et al., 1998; see section “Materials and Methods”), we further determined the *K*_m_ of OsUCH3 to be 185.38 ± 13.04 nM using ubiquitin with 7-amido-4-methylcoumarin (AMC) (Ubiquitin-AMC, human recombinant; BostonBiochem) as the substrate. Accordingly, the *k*_cat_ value was 7.46 s^−1^, and the *k*_cat_/*K*_m_ was 4.0 E + 07 (for a detailed calculation see Supplementary Figure S7 and Supplementary Table S6). This *K*_m_ was similar to that of human UCH-L1 (122 ± 10 nM; Nishikawa et al., 2003), but nearly 10 times higher than that of yeast Yuh1 (20 ± 5 nM; Johnston et al., 1999). Ubiquitin vinyl methyl ester (Ub-VME; LifeSensors) is a specific inhibitor of UCH proteins (Borodovsky et al., 2001). OsUCH3 enzyme activity was completely inhibited in the presence of Ub-VME (Figure 2A and Supplementary Table S7). These results indicate that OsUCH3 is a typical UCH in terms of its activity and inhibition by Ub-VME.

Ubiquitin C-terminal hydrolase proteins can use substrates other than ubiquitin, such as NEDD8 (neural precursor cell expressed developmentally downregulated protein 8; Artavanis-Tsakonas et al., 2010). We therefore examined the deneddylation activity of OsUCH3. As shown in Figure 2C, OsUCH3 had a higher deneddylation activity than the positive control, HsUCHL3. The *K*_m_ of OsUCH3 was 172.70 ± 30.23 nM using NEDD8-AMC (NEDD8-AMC, human recombinant; BostonBiochem) as the substrate (*k*_cat_ = 17.22; *k*_cat_/*K*_m_ = 1.0E + 08; Supplementary Table S7). This activity was also inhibited by Ub-VME and required C96.

### Enzyme Activity of 16 Selected OsUBP Proteins

**FIGURE 3:**
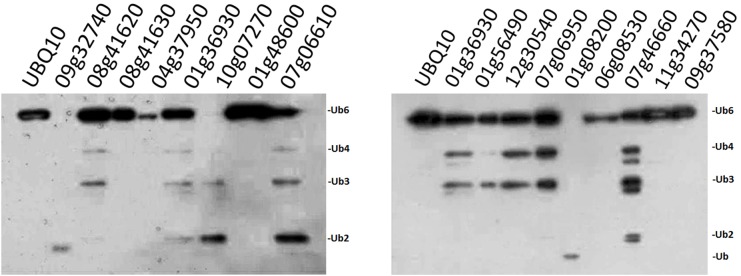
Enzyme activity examination of selected OsUBPs. The enzyme activity of 16 selected OsUBPs (Os09g32740, Os08g41620, Os08g41630, Os04g37950, Os01g36930, Os10g07270, Os01g48600, Os07g06610, Os01g56490, Os12g30540, Os07g06950, Os01g08200, Os06g08530, Os07g46660, Os11g34270, and Os09g37580) were examined *in vivo*, using AtUBQ10 as the substrate. Ten of the OsUBPs (Os09g32740, Os08g41620, Os01g36930, Os10g07270, Os07g06610, Os01g56490, Os12g30540, Os07g06950, Os01g08200, and Os07g46660) showed DUB activity *in vivo*, detected by the degradation of the substrates into small fragments. OsUBP6 (Os01g36930) was used as the positive control.

Since our original purpose was to examine whether the OsUCHs or OsUBPs are involved in stamen development, we selected 16 OsUBPs for use as representatives for the enzymatic analysis of this family because of their expression patterns determined using the GEP (Gene Expression Profiling) (Chen et al., 2015; Supplementary Figure S8). Previously, an enzymatic analysis was only performed for OsUBP6 (Moon et al., 2009). We used an *in vivo* method to examine the enzyme activity of the 16 OsUBPs; however, this activity was only detected in 10 of the 16 selected OsUBPs (Figure 3).

### Two *OsUCH* and Three *OsUBP* Genes Are Preferentially Expressed During Early Stamen Development

**FIGURE 4:**
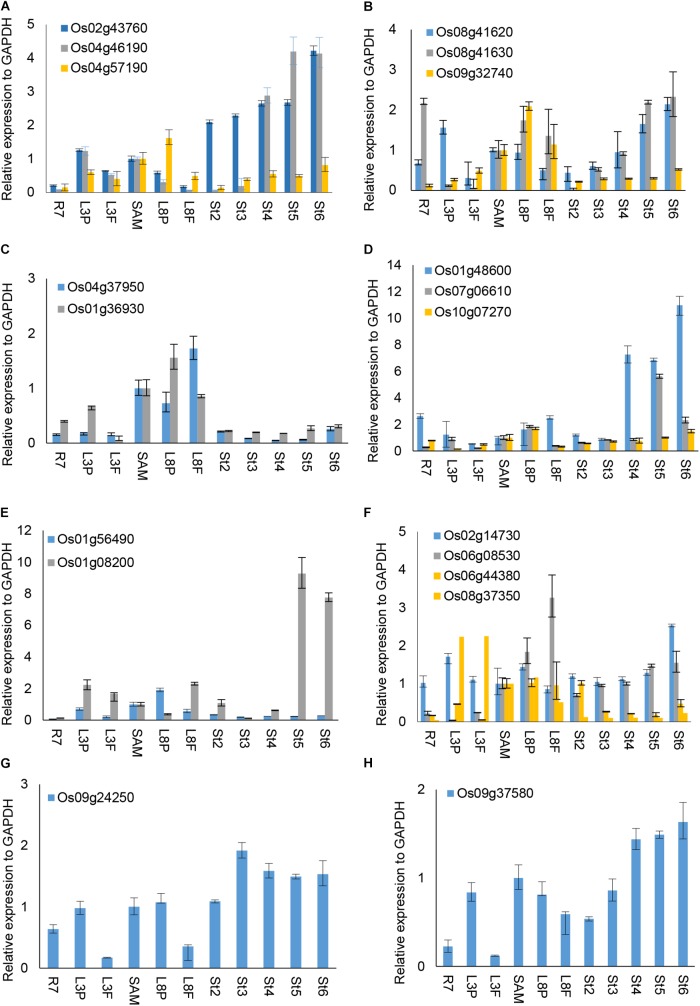
RT-qPCR analysis of selected *OsUCH* and *OsUBP* expression. The selected 3 *OsUCH* and16 *OsUBP* for use as representatives were performed the real-time RT-qPCR analysis because of their expression patterns determined using the GEP (Gene Expression Profiling) and examined the enzyme activity (Chen et al., 2015). Total RNA was prepared from 7-day-old root meristems (R7), shoot tips (ST), the third mature leaf (ML3), the third leaf primordium (LP3), the eighth mature leaf (ML8), the eighth leaf primordium (LP8), and stamens at developmental stages 2–6 (S2–S6), and was subjected to an RT-qPCR analysis. **(A)** *OsUCH* family members, **(B–H)** *OsUBP* family members. The results are the average values obtained from three independent experiments, presented relative to the *GAPDH* expression levels. Error bars indicate SD (*n* = 3).

We were interested to explore whether and how the OsUCH or OsUBP proteins play roles in stamen development. Our previous gene expression profiling revealed various extents of preferential expression of three *OsUCH* and 13 *OsUBP* genes in early stamen development in rice (Supplementary Figure S8). Here, we used real-time RT-qPCR to examine the expression of all five *OsUCH* and 44 selected *OsUBP* genes in rice stamen development from stages 2 to 6 in comparison with those in the shoot apical meristem (SAM) of seedlings with five fully expanded leaves, the root tips of 7-day-old seedlings (R7), and the leaf primordia and newly expanded leaves at the third and eighth nodes, respectively (Supplementary Figure S9). Figure 4 shows the results of the real-time RT-qPCR analysis 3 *OsUCHs* belong to the clade with mouse (*Mus musculus*) *UCHL1* and *UCHL3*, and 16 *OsUBPs* selected for the examination of the enzyme activity (Supplementary Table S8). The real-time RT-PCR revealed that two *OsUCH* genes, Os02g43760 and Os04g46190 (Figure 4A), and three *OsUBP* genes, Os01g48600, Os07g06610 (Figure 4D) and Os01g08200 (Figure 4E) exhibit stamen preferential expression pattern. This information could be useful for future investigations.

**FIGURE 5:**
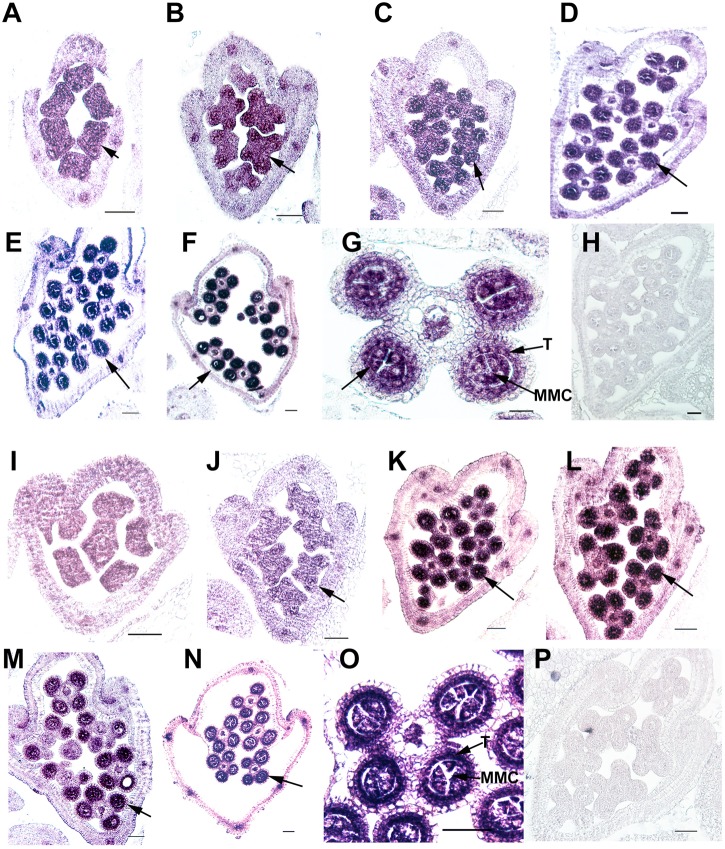
*In situ* hybridization of two *OsUCH* genes during early stamen development in rice. The expression of *OsUCH3* (Os02g43760) **(A–F)** and *OsUCH4* (Os04g46190) **(I–N)** was observed in stamens at developmental stages 4–9 (Sanders et al., 1999). **(G,O)** Figures are magnifications of the expression patterns of *OsUCH3* and *OsUCH4* genes in the meiotic mother cells and tapetum cells. Control sections **(H,P)** hybridized with the sense strand did not show any signal. Arrows indicate the locations of *OsUCH3* and *OsUCH4* expression. Bar = 50 μm. T, tapetum; MMC, microspore mother cell.

Ubiquitin C-terminal hydrolase activity was reported to be involved in gonadal transformation and spermatogenesis, and the stamens are the functionally equivalent plant organ to the male gonads in animals (Bai and Xu, 2013); therefore, we used *in situ* hybridization to examine the expression patterns of two *OsUCHs*, Os02g43760 and Os04g46190 (Figure 5), and seven *OsUBPs* (Supplementary Figure S10) during stamen development, because of their high expression levels in the mature stamen. Consistent with the results from the microarray and RT-qPCR analysis, the expression levels of the two *OsUCH* genes were not only highly upregulated in stamen development, but were also concentrated in the meiotic mother cells and tapetum cells (Figure 5). This expression pattern suggests that the two *OsUCH* genes may play roles in germ cell induction and/or differentiation in rice stamens.

Of the 16 *OsUBP* genes investigated, eight exhibited stamen-preferential expression patterns (Supplementary Table S8, column 4), and an *in situ* hybridization analysis confirmed these expression patterns for five of the eight genes (Supplementary Table S8, column 5). Among these five, three genes encode proteins exhibiting the enzyme activities (Os08g41620, Os07g06610, and Os01g08200). Although the enzyme activity of the proteins encoded by Os09g32740, Os01g36930, Os10g07270, and Os01g56490 were detected, the stamen-preferential expression patterns of these genes revealed by the GEP were not confirmed using RT-qPCR.

Taken together, a few conclusions can be made from this work. First, we clarified that there are five *OsUCH* and 44 *OsUBP* genes present in the rice genome, and established their phylogenetic relationships based on their sequence similarities. Second, we examined the enzymatic activities of all five OsUCH and 16 representative OsUBP proteins, revealing that four of the five OsUCH and 10 of the 16 OsUBP proteins have DUB activity. In addition, the detailed enzymatic features of OsUCH3 were characterized. The expression patterns of all five *OsUCH* and 44 *OsUBP* genes were examined, revealing that two *OsUCH* and seven *OsUBP* genes were preferentially expressed in the early stages of stamen development in rice. This work systematically clarified the previously conflicting information about this important gene family and laid down a reliable bioinformatic base for the future investigation of genes in this family, particularly for their potential roles in rice stamen development.

## Author Contributions

D-HW performed most of the experiments involving RNAi, mutant characterization, RT-qPCR analyses, and *in situ* hybridization. WS performed the enzymatic activity assays and the associated vector construction. S-WW and Y-FZ are involved in the computational analysis and constructed the phylogenetic tree of the UCH and UBP protein families. Z-SC calculated the kinetic parameters. J-DH and H-TZ performed the bioinformatic analysis. Y-MQ provided the methods hand participated in analyzing the enzymatic activity. J-CL helped with the phylogenetic tree construction. Z-HX and S-NB designed most of the experiments. S-NB wrote the article.

## Conflict of Interest Statement

The authors declare that the research was conducted in the absence of any commercial or financial relationships that could be construed as a potential conflict of interest.

## Footnotes

1 http://merops.sanger.ac.uk/

2 https://www.ncbi.nlm.nih.gov/Structure/cdd/wrpsb.cgi?INPUT_TYPE=live&SEQUENCE=BAG95328.1

3 http://www.ncbi.nlm.nih.gov/protein

4 https://shigen.nig.ac.jp/rice/oryzabase/

5 http://www.gramene.org/

6 https://www.ebi.ac.uk/merops/
