# Supplementary material for: Characterization of the Ubiquitin C-Terminal Hydrolase and Ubiquitin-Specific Protease Families in Rice (Oryza sativa)
Source: Front Plant Sci. 2018 Nov 15;9:1636. doi: 10.3389/fpls.2018.01636 (PMC6249995; doi:10.3389/fpls.2018.01636)
Supplement: Supplementary file 1 [file Image_1.pdf]

Figure S1 Phylogeny of the UCH protein family. OsUCH phylogenetic analysis was based on 60 protein sequences of the members of OsUCH family, retrieved from the NCBI website (http://www.ncbi.nlm.nih.gov/protein, See the supplemental table for the gene number.) and UniProt database of 13 species, including *Saccharomyces cerevisiae (Sce), Schizosaccharomyces pombe (Spo), Physcomitrella patens (ppa), Selaginella moellendorffii (Smo), Zea mays (zm), Oryza sativa (osm), Sorghum bicolor (Sbi), Arabidopsis thaliana (ath), Populus trichocarpa (pop), Monopterus albus (moa), Mus musculus (mou), Rattus norregicus (rat), Homo sapiens (hum).* The right figure was the magnified image showed the genes from sbi/C5YA10 to zm/C0HHI4 including osm4g57190, osm4g46190 and osm2g43760.

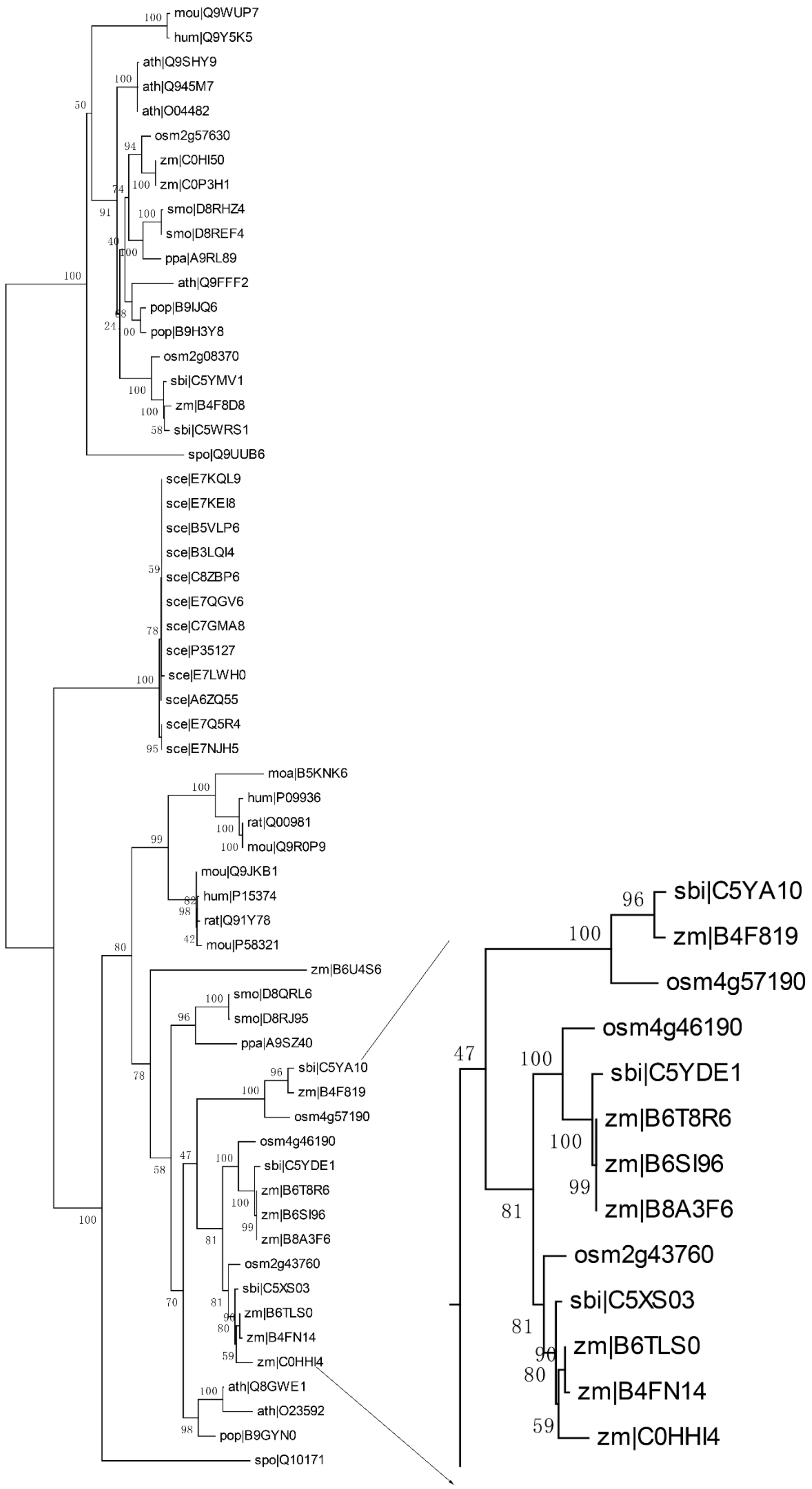
mou|Q9WUP7
hum|Q9Y5K5
ath|Q9SHY9
ath|Q945M7
ath|O04482
osm2g57630
zm|C0HI50
zm|C0P3H1
smo|D8RHZ4
smo|D8REF4
ppa|A9RL89
ath|Q9FFF2
pop|B9IJQ6
pop|B9H3Y8
osm2g08370
sbi|C5YMV1
zm|B4F8D8
sbi|C5WRS1
spo|Q9UUB6
sce|E7KQL9
sce|E7KEI8
sce|B5VLP6
sce|B3LQI4
sce|C8ZBP6
sce|E7QGV6
sce|C7GMA8
sce|P35127
sce|E7LWH0
sce|A6ZQ55
sce|E7Q5R4
sce|E7NJH5
moa|B5KNK6
hum|P09936
rat|Q00981
mou|Q9R0P9
mou|Q9JKB1
hum|P15374
rat|Q91Y78
mou|P58321
zm|B6U4S6
smo|D8QRL6
smo|D8RJ95
ppa|A9SZ40
sbi|C5YA10
zm|B4F819
osm4g57190
osm4g46190
sbi|C5YDE1
zm|B6T8R6
zm|B6SI96
zm|B8A3F6
osm2g43760
sbi|C5XS03
zm|B6TLS0
zm|B4FN14
zm|C0HHI4
ath|Q8GWE1
ath|O23592
pop|B9GYN0
spo|Q10171

Figure S2 Phylogeny of the UBP protein family. The OsUBPs phylogenetic analysis was based on 202 protein sequences of the members of OsUBP family, retrieved from UniProt database of 12 species, including *Saccharomyces cerevisiae (Sce), Schizosaccharomyces pombe (Spo), Physcomitrella patens (ppa), Selaginella moellendorffii (Smo), Zea mays (zm), Oryza sativa (osm), Sorghum bicolor (Sbi), Arabidopsis thaliana (ath), Populus trichocarpa (pop), Mus musculus (mou), Rattus norregicus (rat), Homo sapiens (hum).* See the supplemental table for the gene number.

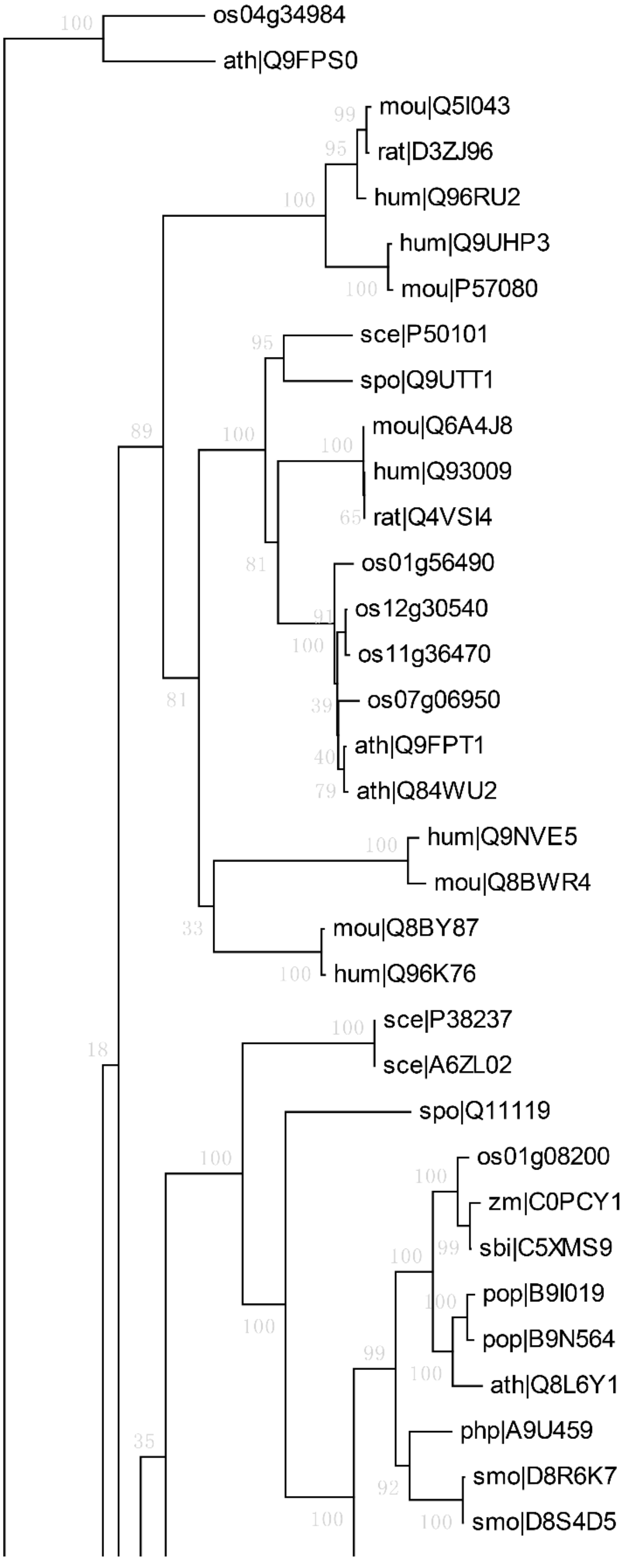
os04g34984
ath|Q9FPS0
mou|Q5I043
rat|D3ZJ96
hum|Q96RU2
hum|Q9UHP3
mou|P57080
sce|P50101
spo|Q9UTT1
mou|Q6A4J8
hum|Q93009
rat|Q4VSI4
os01g56490
os12g30540
os11g36470
os07g06950
ath|Q9FPT1
ath|Q84WU2
hum|Q9NVE5
mou|Q8BWR4
mou|Q8BY87
hum|Q96K76
sce|P38237
sce|A6ZL02
spo|Q11119
os01g08200
zm|C0PCY1
sbi|C5XMS9
pop|B9I019
pop|B9N564
ath|Q8L6Y1
php|A9U459
smo|D8R6K7
smo|D8S4D5

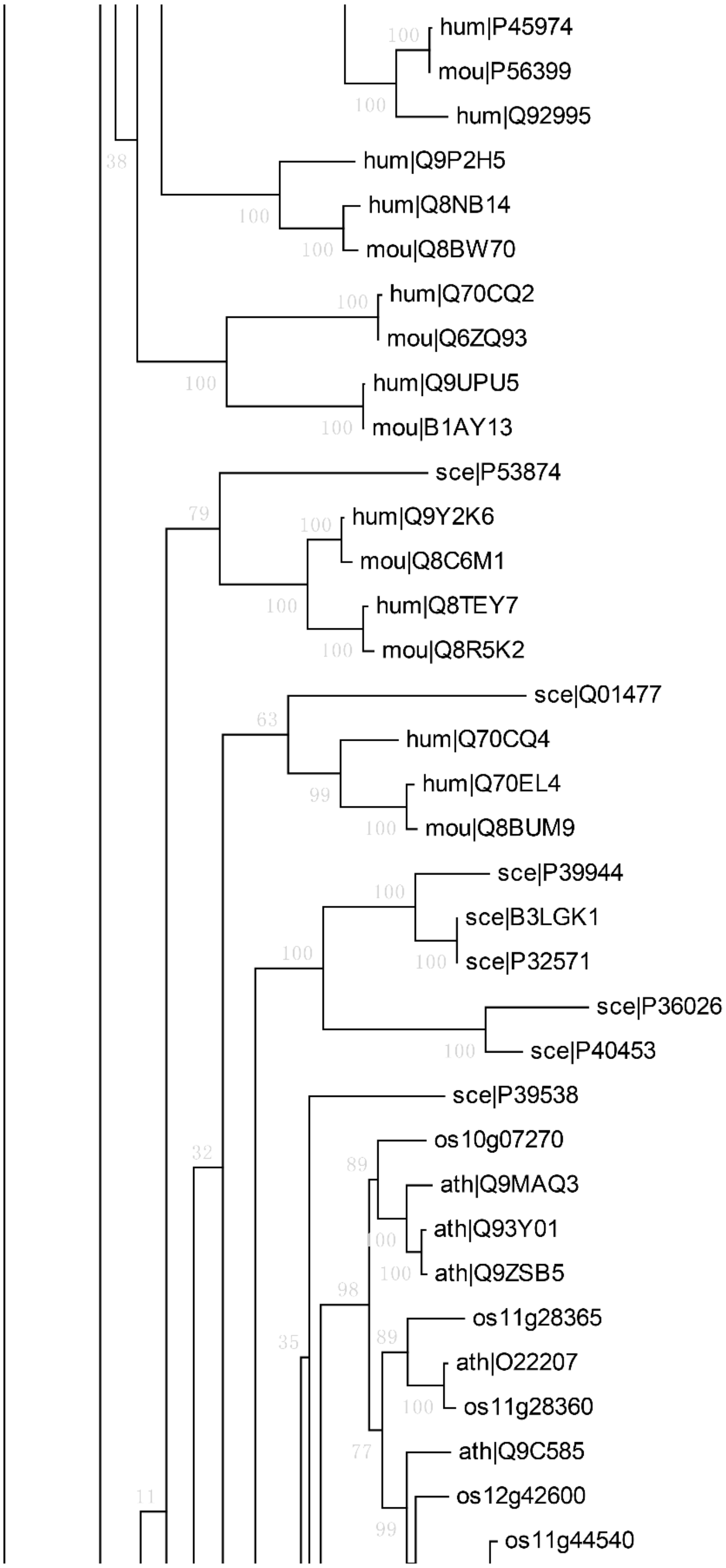
hum|P45974
mou|P56399
hum|Q92995
hum|Q9P2H5
hum|Q8NB14
mou|Q8BW70
hum|Q70CQ2
mou|Q6ZQ93
hum|Q9UPU5
mou|B1AY13
sce|P53874
hum|Q9Y2K6
mou|Q8C6M1
hum|Q8TEY7
mou|Q8R5K2
sce|Q01477
hum|Q70CQ4
hum|Q70EL4
mou|Q8BUM9
sce|P39944
sce|B3LGK1
sce|P32571
sce|P36026
sce|P40453
sce|P39538
os10g07270
ath|Q9MAQ3
ath|Q93Y01
ath|Q9ZSB5
os11g28365
ath|O22207
os11g28360
ath|Q9C585
os12g42600
os11g44540

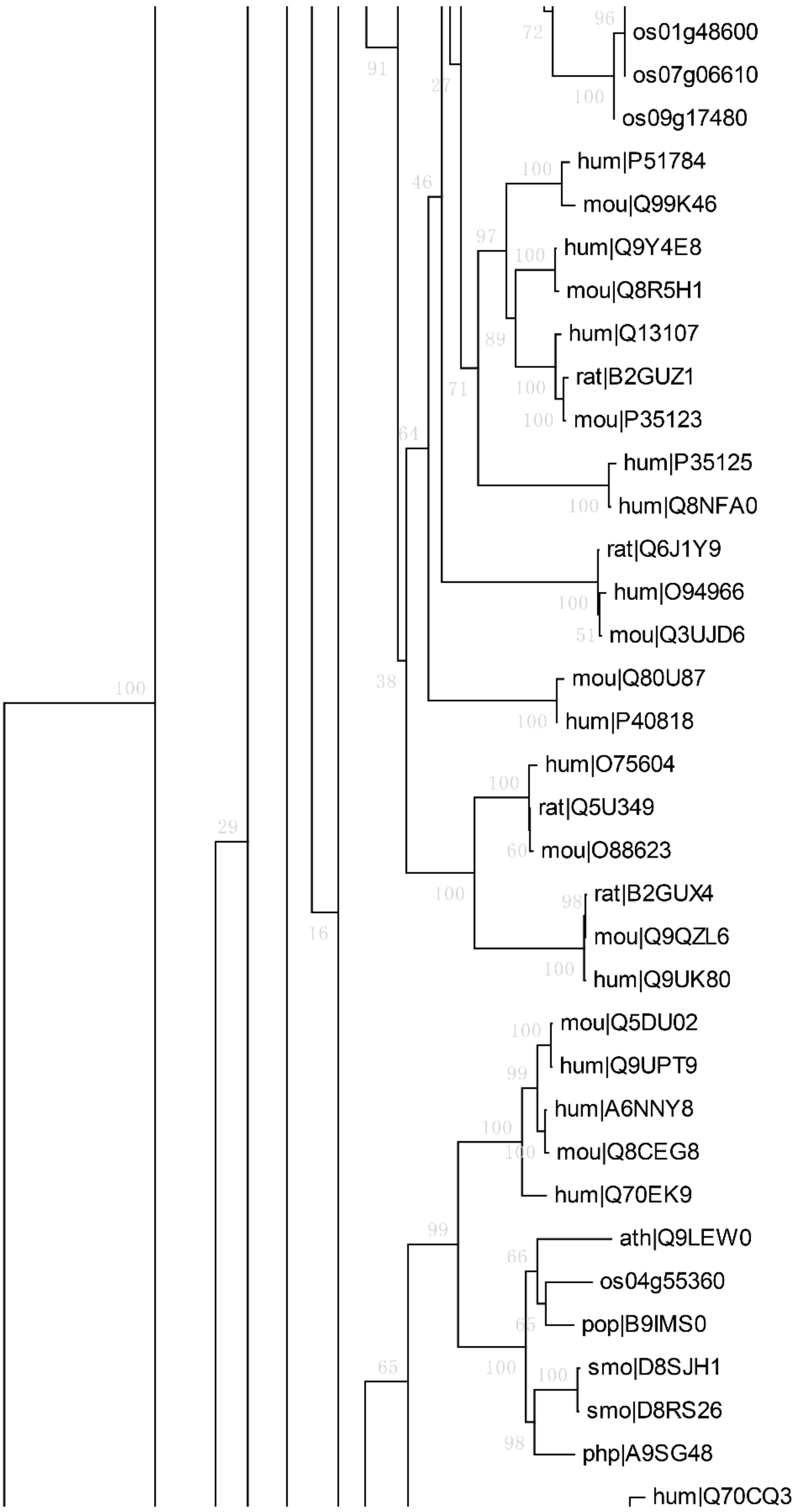
os01g48600
os07g06610
os09g17480
hum|P51784
mou|Q99K46
hum|Q9Y4E8
mou|Q8R5H1
hum|Q13107
rat|B2GUZ1
mou|P35123
hum|P35125
hum|Q8NFA0
rat|Q6J1Y9
hum|O94966
mou|Q3UJD6
mou|Q80U87
hum|P40818
hum|O75604
rat|Q5U349
mou|O88623
rat|B2GUX4
mou|Q9QZL6
hum|Q9UK80
mou|Q5DU02
hum|Q9UPT9
hum|A6NNY8
mou|Q8CEG8
hum|Q70EK9
ath|Q9LEW0
os04g55360
pop|B9IMS0
smo|D8SJH1
smo|D8RS26
php|A9SG48
hum|Q70CQ3

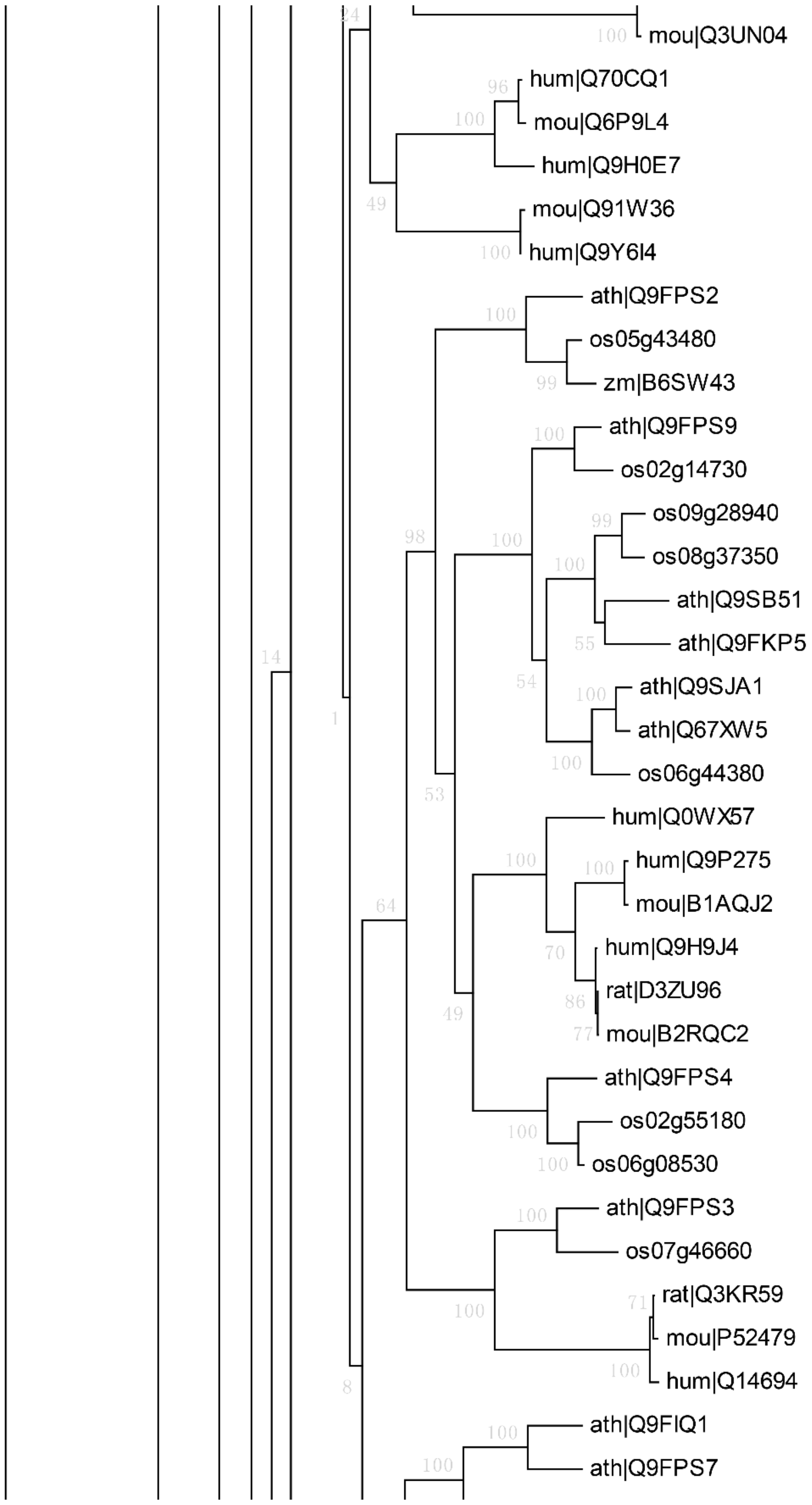
mou|Q3UN04
hum|Q70CQ1
mou|Q6P9L4
hum|Q9H0E7
mou|Q91W36
hum|Q9Y6I4
ath|Q9FPS2
os05g43480
zm|B6SW43
ath|Q9FPS9
os02g14730
os09g28940
os08g37350
ath|Q9SB51
ath|Q9FKP5
ath|Q9SJA1
ath|Q67XW5
os06g44380
hum|Q0WX57
hum|Q9P275
mou|B1AQJ2
hum|Q9H9J4
rat|D3ZU96
mou|B2RQC2
ath|Q9FPS4
os02g55180
os06g08530
ath|Q9FPS3
os07g46660
rat|Q3KR59
mou|P52479
hum|Q14694
ath|Q9FIQ1
ath|Q9FPS7

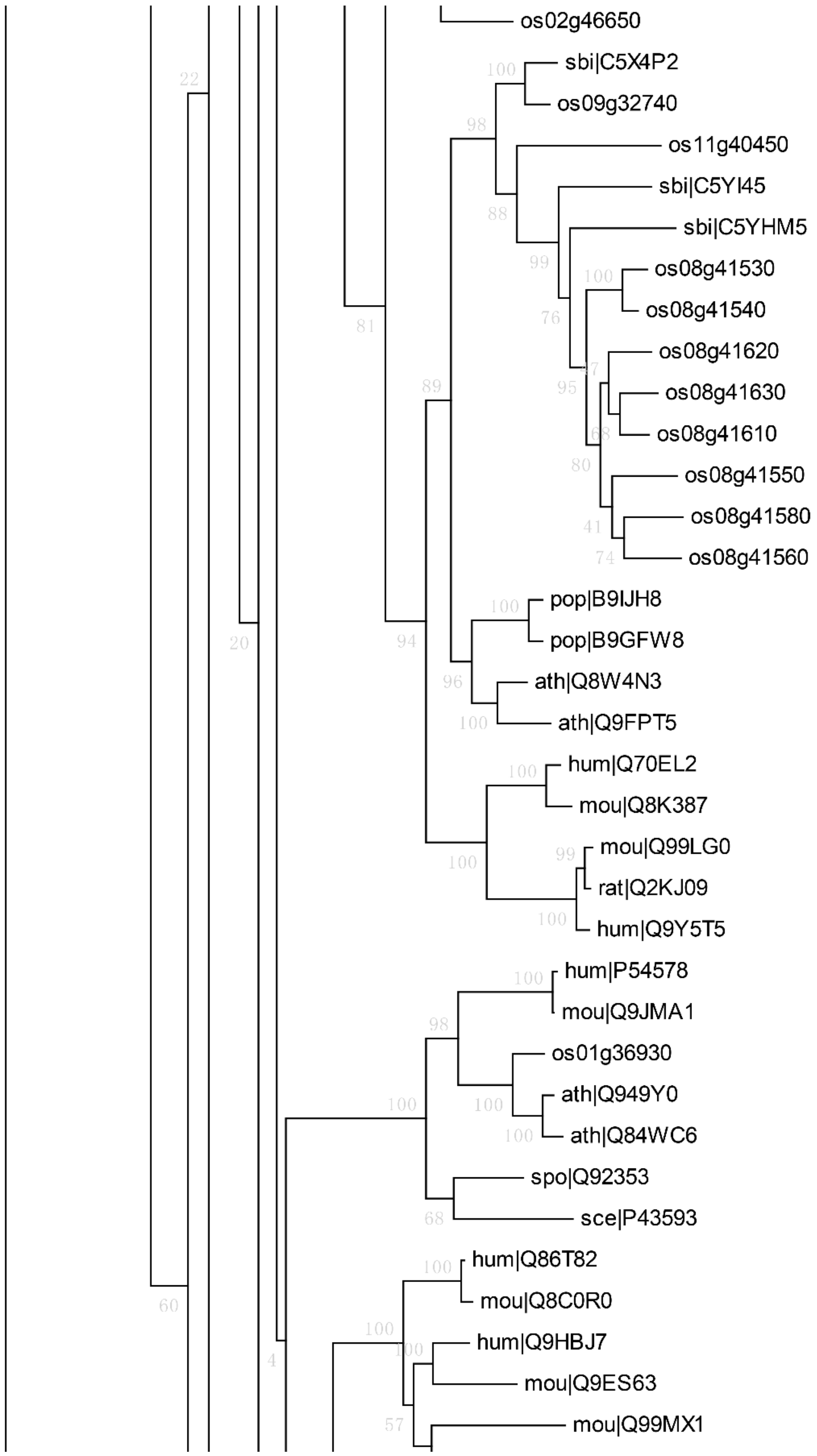
os02g46650
sbi|C5X4P2
os09g32740
os11g40450
sbi|C5YI45
sbi|C5YHM5
os08g41530
os08g41540
os08g41620
os08g41630
os08g41610
os08g41550
os08g41580
os08g41560
pop|B9IJH8
pop|B9GFW8
ath|Q8W4N3
ath|Q9FPT5
hum|Q70EL2
mou|Q8K387
mou|Q99LG0
rat|Q2KJ09
hum|Q9Y5T5
hum|P54578
mou|Q9JMA1
os01g36930
ath|Q949Y0
ath|Q84WC6
spo|Q92353
sce|P43593
hum|Q86T82
mou|Q8C0R0
hum|Q9HBJ7
mou|Q9ES63
mou|Q99MX1
22
100
98
88
99
100
76
95
80
41
74
81
89
94
20
100
96
100
100
100
99
100
100
98
100
100
100
68
100
60
100
4
57

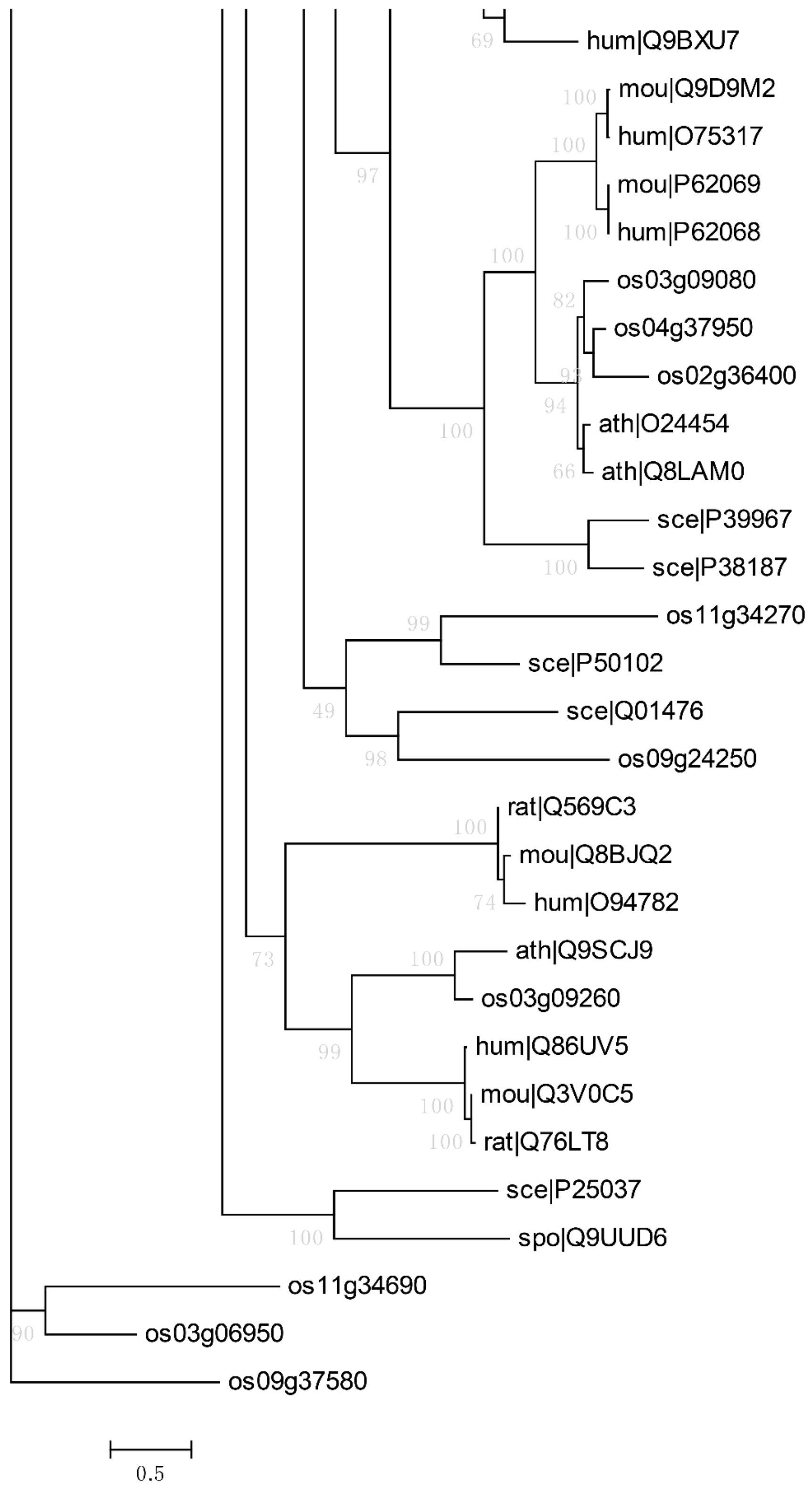
hum|Q9BXU7
mou|Q9D9M2
hum|O75317
mou|P62069
hum|P62068
os03g09080
os04g37950
os02g36400
ath|O24454
ath|Q8LAM0
sce|P39967
sce|P38187
os11g34270
sce|P50102
sce|Q01476
os09g24250
rat|Q569C3
mou|Q8BJQ2
hum|O94782
ath|Q9SCJ9
os03g09260
hum|Q86UV5
mou|Q3V0C5
rat|Q76LT8
sce|P25037
spo|Q9UUD6
os11g34690
os03g06950
os09g37580
0.5

Figure S3 Model of the 3D structure of OsUCH3 generated using SWISS-MODEL (http://swissmodel.expasy.org). The template of this model is AtUCH3 (1xd3A) protein. GenBank Accession Nos.: *AtUCH3* (At4g17510).

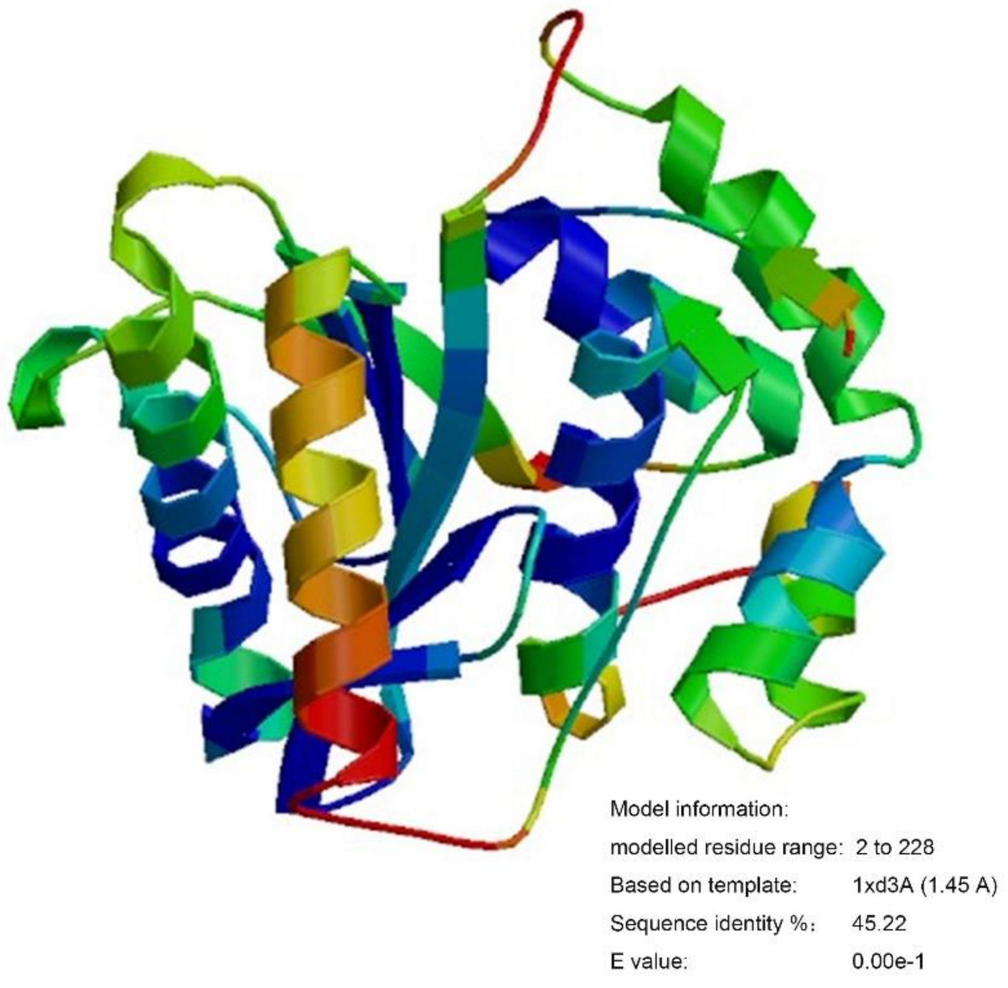

Figure S4 Sequence alignment of OsUCH3 with other closely related UCH proteins from different species. ClustalX alignment of Arabidopsis UCHs and rice OsUCH3 protein sequences. Amino acid residues displaying 100% identity or similarity in the two homologs are shaded clours. Asterisk (*) indicates the conserved active Cys site which is critical for the enzyme activity. Dashes represent gaps that were introduced to maximize alignment. GenBank Accession Nos.: *AtUCH1* (At5g16310), *AtUCH2* (At1g65650), *AtUCH3* (At4g17510), AK066320 (Os02g43760), AK067359 (Os02g08370), AK058380 (Os02g57630), AK103595 (Os04g57190), AK059677 (Os04g46190).

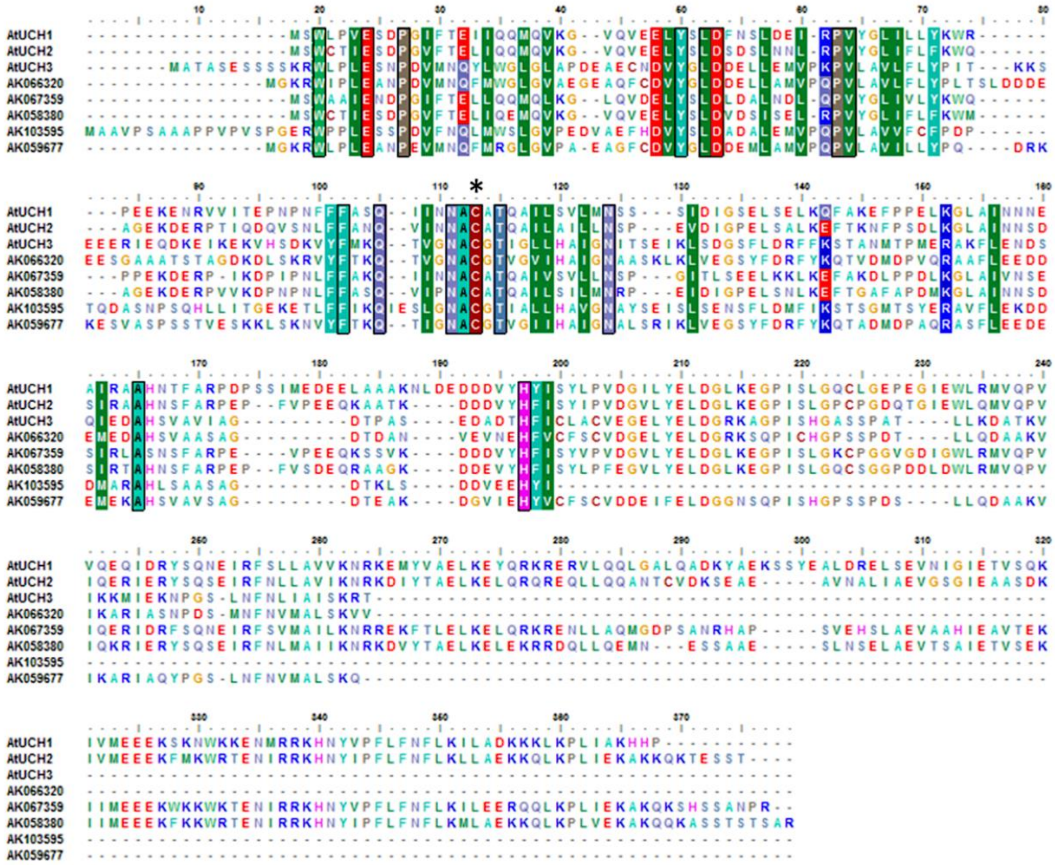

Figure S5 Experimental design of site-directed mutagenesis. According to the protein sequence alignment, Cys96 is the possible critical active site of OsUCH3. Using the Overlap-PCR method according as to Williams (Williams et al., 2014), we designed two pairs of primers. The primer pair F1 and R1 can amplify the whole gene of *OsUCH3*. The mutated primers F2 and R2 are designed to have the mutated nucleotide sequence as showed in Supplemental Table ST1 So that, using F1 and R2, the first half mutated *OsUCH3* gene can be amplified, while using F2 and R1, the last half mutated *OsUCH3* gene can be amplified. Finally, together using the two mixed half mutated genes as template, with the help of F1 and R1, the whole mutated *OsUCH3* gene can be amplified.

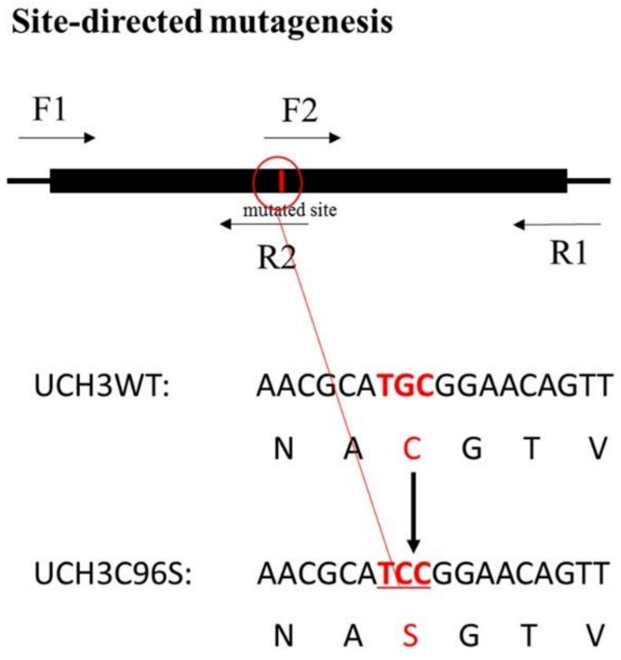

Figure S6 Circular dichroism spectrometer of OsUCH3 and the OsUCH3^{C96S} mutant protein. In order to avoid the effect of protein labeling on the secondary structure as much as possible, we used the pETDuet-1 vector to carry out prokaryotic expression of OsUCH3 and OsUCH3^{C96S} protein so that the protein contained only a 6x His tag. The circular dichroism absorption at the same concentration of OsUCH3 and OsUCH3^{C96S} prokaryotic expression protein at 190 nm to 250 nm was examined using a circular dichroism spectrometer. The results showed that there was no significant secondary structure change. OsUCH3 activity site mutation did not affect the change of its secondary structure

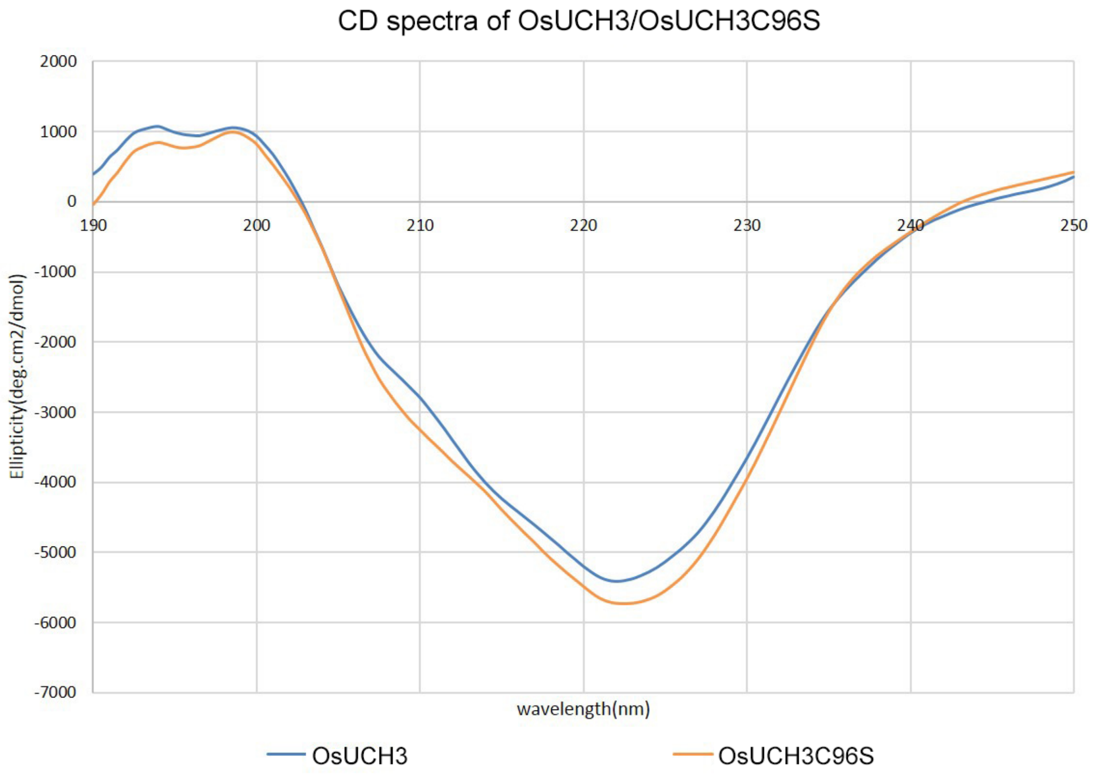

Figure S7 Calculation process for kinetic parameters. Detail assay process see methods. Michaelis constant Km is the substrate concentration at which the reaction rate is at half-maximum, measuring the substrate's affinity for the enzyme. [E] is the enzyme concentration. K_{cat} is catalytic rate of product formation. K_{cat}/K_m is calculated for measuring how efficient for an enzyme coverts a substrates into product. (Dang et al., 1998)

Michaelis-Menten kinetics:

$$V_0 = V_{max}\frac{[S]}{K_m + [S]}$$

Enzyme concentration:

$$[E] = \frac{\mathrm{m}}{\mathrm{M}}$$

Catalytic constant:

$$K_{cat} = \frac{V_{max}}{[\mathrm{E}]}$$

Figure S8 Expression patterns of *OsUCH* and *OsUBP* genes in early stamen development, determined from the whole genome dynamic gene expression profile (GEP) of rice (Chen et al., 2015). 1-14 indicate the corresponding UBP gene group 1-14 respectively; 15 indicates the UCH genes. LP: leaf primordia; ML: mature leaves; S2-S5: stages of stamen development.

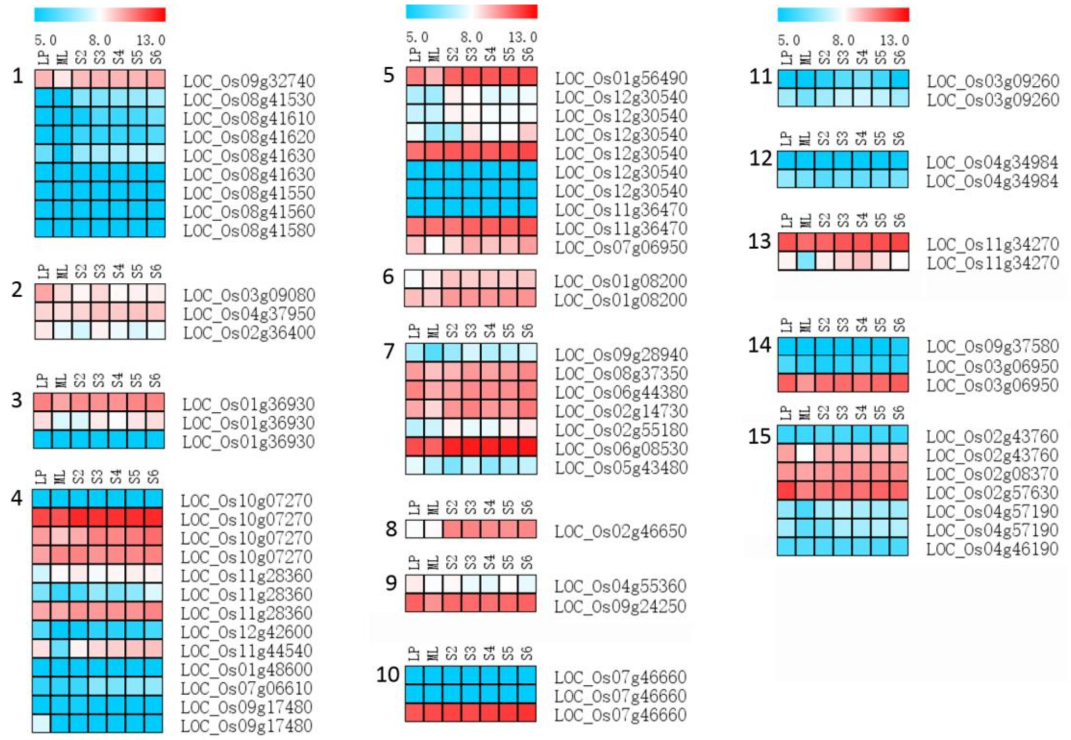

Figure 9 RT-qPCR analysis of all *OsUCH* and *OsUBP* expression. Total RNA was prepared from seven-day-old root meristems (R7), shoot tips (ST), the third mature leaf (ML3), the third leaf primordium (LP3), the eighth mature leaf (ML8), the eighth leaf primordium (LP8), and stamens at developmental stages 2–6 (S2–S6), and was subjected to an RT-qPCR analysis. (a) *OsUCH* family members, (b–h) *OsUBP* family members. The results are the average values obtained from three independent experiments, presented relative to the *GAPDH* expression levels. Error bars indicate SD (n = 3).

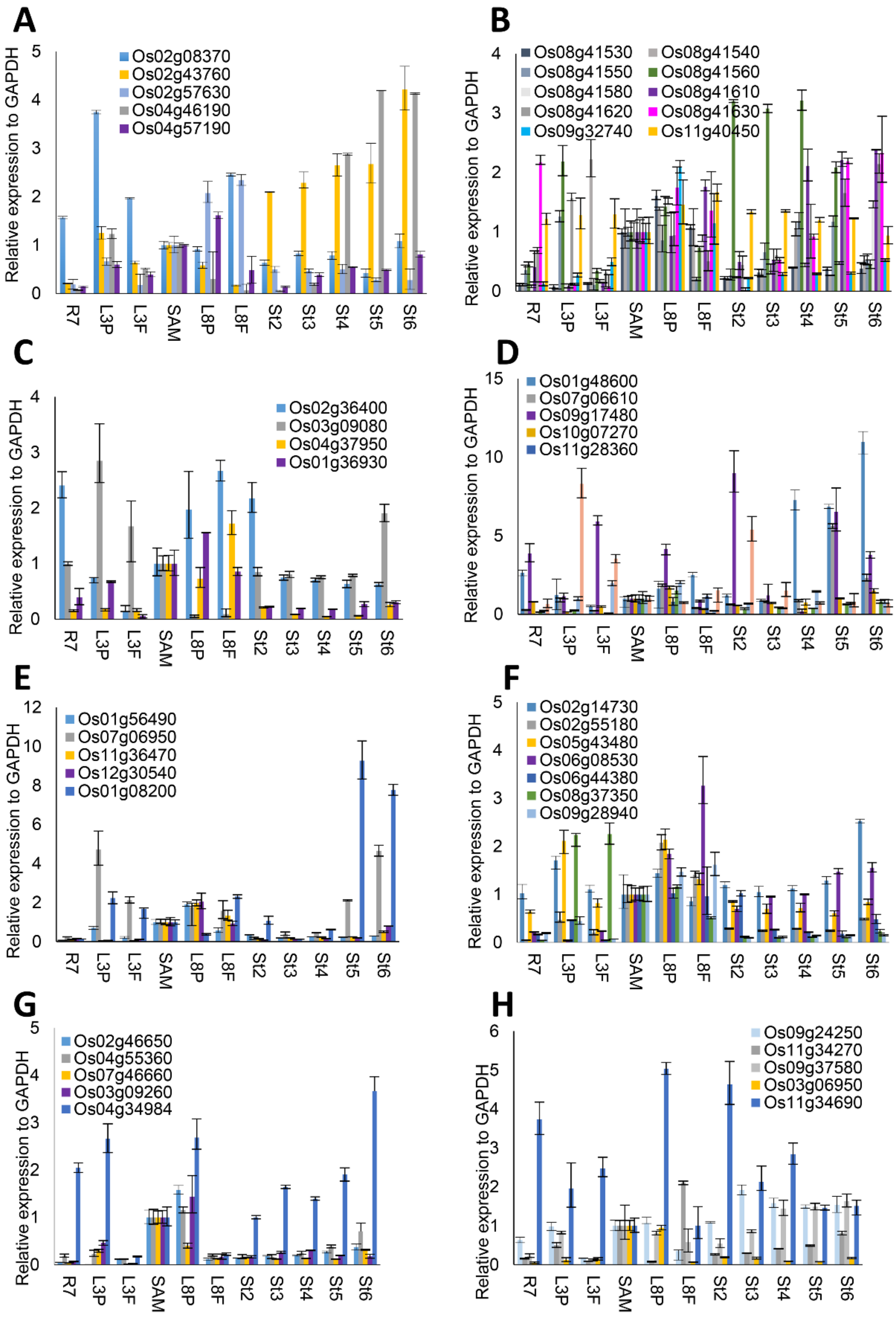
A
Relative expression to GAPDH
Os02g08370
Os02g43760
Os02g57630
Os04g46190
Os04g57190
B
Relative expression to GAPDH
Os08g41530
Os08g41540
Os08g41550
Os08g41560
Os08g41580
Os08g41610
Os08g41620
Os08g41630
Os09g32740
Os11g40450
C
Relative expression to GAPDH
Os02g36400
Os03g09080
Os04g37950
Os01g36930
D
Relative expression to GAPDH
Os01g48600
Os07g06610
Os09g17480
Os10g07270
Os11g28360
E
Relative expression to GAPDH
Os01g56490
Os07g06950
Os11g36470
Os12g30540
Os01g08200
F
Relative expression to GAPDH
Os02g14730
Os02g55180
Os05g43480
Os06g08530
Os06g44380
Os08g37350
Os09g28940
G
Relative expression to GAPDH
Os02g46650
Os04g55360
Os07g46660
Os03g09260
Os04g34984
H
Relative expression to GAPDH
Os09g24250
Os11g34270
Os09g37580
Os03g06950
Os11g34690
R7
L3P
L3F
SAM
L8P
L8F
St2
St3
St4
St5
St6

Figure S10 *In situ* hybridization analysis of selected *OsUBP* genes during rice early stamen development. G1 is group1, G2 is group2, G4 is group4 and G7 is group7 according as Fig.3. Control sections (C) hybridized with the sense strand did not show any signal. Arrows indicate the genes expression positions. Bar = 50 μm.

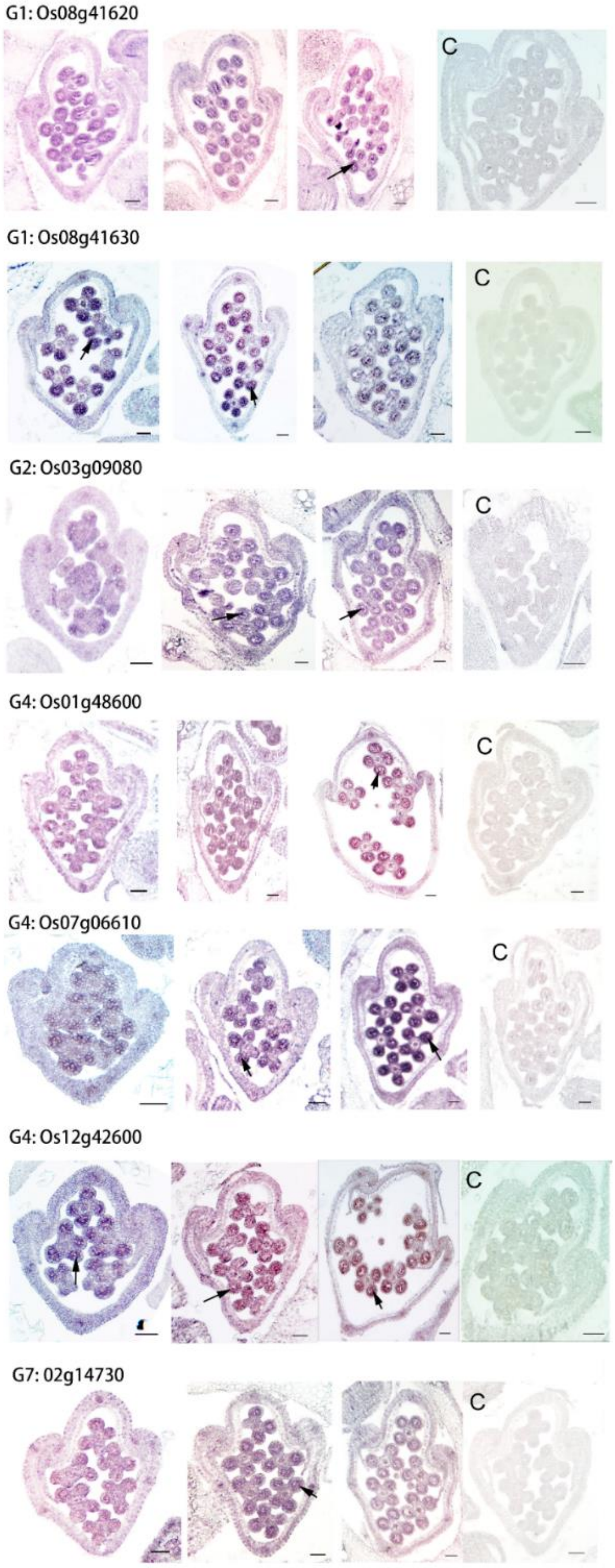
G1: Os08g41620
C
G1: Os08g41630
C
G2: Os03g09080
C
G4: Os01g48600
C
G4: Os07g06610
C
G4: Os12g42600
C
G7: 02g14730
C
