# Supplementary material for: Characterization of the Ubiquitin C-Terminal Hydrolase and Ubiquitin-Specific Protease Families in Rice (Oryza sativa)
Source: Front Plant Sci. 2018 Nov 15;9:1636. doi: 10.3389/fpls.2018.01636 (PMC6249995; doi:10.3389/fpls.2018.01636)
Supplement: Supplementary file 2 [file Table_1.pdf]

Table S1 The *OsUCH* and *OsUBP* genes from NCBI.

	Accession Number	gene	Protein length	annotation
1	Accession: BAD82061.1 GI: 56785209	Os01g56490	1108 aa protein	putative ubiquitin carboxyl-terminal hydrolase 7 [Oryza sativa Japonica Group]
2	Accession: BAB56080.1 GI: 14209584	Os01g56490	1108 aa protein	putative ubiquitin carboxyl-terminal hydrolase 7 [Oryza sativa Japonica Group]
3	Accession: EEC71554.1 GI: 218189127	Os01g56490	1075 aa protein	hypothetical protein OsI_03906 [Oryza sativa Indica Group]
4	Accession: BAF06299.2 GI: 255673725	Os01g56490	230 aa protein	Os01g0771400, partial [Oryza sativa Japonica Group]
5	Accession: NP_001044385.2 GI: 297597686	Os01g56490	230 aa protein	Os01g0771400, partial [Oryza sativa Japonica Group]
6	Accession: CBC76809.1 GI: 257723989	Os01g56490	1108 aa protein	unnamed protein product [Oryza sativa Japonica Group]
7	Accession: CAW79173.1 GI: 219894295	Os01g56490	1108 aa protein	unnamed protein product [Oryza sativa Japonica Group]
8	Accession: Q8LQ36.1 GI: 75157438	Os01g63250	336 aa protein	RecName: Full=Putative ataxin-3 homolog [Oryza sativa Japonica Group]
9	Accession: BAD28018.1 GI: 50252088	Os02g08370	329 aa protein	putative ubitquitin C-terminal hydrolase [Oryza sativa Japonica Group]
10	Accession: EEC73821.1 GI: 218191394	Os02g46650	672 aa protein	hypothetical protein OsI_08545 [Oryza sativa Indica Group]
11	Accession: EEE57611.1 GI: 222623479	Os02g46650	672 aa protein	hypothetical protein OsJ_08002 [Oryza sativa Japonica Group]
12	Accession: BAF09715.1 GI: 113537332	Os02g46650	257 aa protein	Os02g0693400 [Oryza sativa Japonica Group]
13	Accession: NP_001047801.1 GI: 115448043	Os02g46650	257 aa protein	Os02g0693400 [Oryza sativa Japonica Group]
14	Accession: BAD23124.1 GI: 48716519	Os02g57630	331 aa protein	putative ubiquitin C-terminal hydrolase [Oryza sativa Japonica Group]
15	Accession: BAD22892.1 GI: 48716277	Os02g57630	331 aa protein	putative ubitquitin C-terminal hydrolase [Oryza sativa Japonica Group]
16	Accession: ABF94151.1 GI: 108706356	Os03g06950	1579 aa protein	Ubiquitin carboxyl-terminal hydrolase family protein, expressed [Oryza sativa Japonica Group]

17	Accession: BAF10983.1 GI: 113547540	Os03g06950	505 aa protein	Os03g0165600, partial [Oryza sativa Japonica Group]
18	Accession: EEC74581.1 GI: 218192154	Os03g06950	1598 aa protein	hypothetical protein OsI_10153 [Oryza sativa Indica Group]
19	Accession: EEE58383.1 GI: 222624251	Os03g06950	1601 aa protein	hypothetical protein OsJ_09540 [Oryza sativa Japonica Group]
20	Accession: NP_001049069.1 GI: 115450937	Os03g06950	505 aa protein	Os03g0165600, partial [Oryza sativa Japonica Group]
21	Accession: A3AF13.2 GI: 152112340	Os03g09260	1079 aa protein	Ubiquitin-specific-processing protease 26 [Oryza sativa Japonica Group]
22	Accession: A2XDG4.1 GI: 152112339	Os03g09260	1079 aa protein	Ubiquitin-specific-processing protease 26 [Oryza sativa Indica Group]
23	Accession: BAF11157.2 GI: 255674273	Os03g09260	311 aa protein	Os03g0192800 [Oryza sativa Japonica Group]
24	Accession: NP_001049243.2 GI: 297600463	Os03g09260	311 aa protein	Os03g0192800 [Oryza sativa Japonica Group]
25	Accession: ABF94422.1 GI: 108706627	Os03g09260	311 aa protein	ubiquitin-specific protease 26, putative, expressed [Oryza sativa Japonica Group]
26	Accession: CAH66407.1 GI: 116309321	Os04g34984	538 aa protein	OSIGBa0093L02.3 [Oryza sativa Indica Group]
27	Accession: BAH92668.1 GI: 255675466	Os04g34984	145 aa protein	Os04g0429200 [Oryza sativa Japonica Group]
28	Accession: NP_001173940.1 GI: 297723153	Os04g34984	145 aa protein	Os04g0429200 [Oryza sativa Japonica Group]
29	Accession: EEC77331.1 GI: 218194904	Os04g35900	429 aa protein	hypothetical protein OsI_16005 [Oryza sativa Indica Group]
30	Accession: EEE61055.1 GI: 222628923	Os04g35900	429 aa protein	hypothetical protein OsJ_14912 [Oryza sativa Japonica Group]
31	Accession: CAH66845.1 GI: 116309806	Os04g35900	429 aa protein	H0525C06.8 [Oryza sativa Indica Group]
32	Accession: BAF14785.1 GI: 113564442	Os04g35900	429 aa protein	Os04g0439900 [Oryza sativa Japonica Group]
33	Accession: NP_001052871.1 GI: 115458542	Os04g35900	429 aa protein	Os04g0439900 [Oryza sativa Japonica Group]
34	Accession: BAG93784.1 GI: 215704350	Os04g35900	429 aa protein	unnamed protein product [Oryza sativa Japonica Group]

35	Accession: EAZ32198.1 GI: 125591848	Os04g55360	562 aa protein	hypothetical protein OsJ_16406 [Oryza sativa Japonica Group]
36	Accession: CAH68425.1 GI: 90399346	Os04g55360	562 aa protein	H0811D08.10 [Oryza sativa Indica Group]
37	Accession: BAF15984.2 GI: 255675832	Os04g55360	591 aa protein	Os04g0647300, partial [Oryza sativa Japonica Group]
38	Accession: NP_001054070.2 GI: 297603462	Os04g55360	591 aa protein	Os04g0647300, partial [Oryza sativa Japonica Group]
39	Accession: BAG94856.1 GI: 215704828	Os04g55360	370 aa protein	unnamed protein product [Oryza sativa Japonica Group]
40	Accession: CAE03179.2 GI: 38344011	Os04g55360	370 aa protein	OSJNBa0070O11.10 [Oryza sativa Japonica Group]
41	Accession: EEE64318.1 GI: 222632186	Os05g43480	628 aa protein	hypothetical protein OsJ_19155 [Oryza sativa Japonica Group]
42	Accession: BAG95790.1 GI: 215736861	Os06g08530	612 aa protein	unnamed protein product [Oryza sativa Japonica Group]
43	Accession: BAC15941.1 GI: 22831079	Os07g06610	951 aa protein	putative ubiquitin C-terminal hydrolase [Oryza sativa Japonica Group]
44	Accession: BAF20850.2 GI: 255677531	Os07g06610	911 aa protein	Os07g0160000 [Oryza sativa Japonica Group]
45	Accession: NP_001058936.2 GI: 297606766	Os07g06610	911 aa protein	Os07g0160000 [Oryza sativa Japonica Group]
46	Accession: EEE66613.1 GI: 222636481	Os07g06950	1017 aa protein	hypothetical protein OsJ_23193 [Oryza sativa Japonica Group]
47	Accession: EEE55453.1 GI: 222619321	Os07g06950	1075 aa protein	hypothetical protein OsJ_03614 [Oryza sativa Japonica Group]
48	Accession: EEC81563.1 GI: 218199136	Os07g06950	1089 aa protein	hypothetical protein OsI_25004 [Oryza sativa Indica Group]
49	Accession: BAC83609.1 GI: 34395211	Os07g06950	1116 aa protein	putative ubiquitin-specific protease [Oryza sativa Japonica Group]
50	Accession: CBC76814.1 GI: 257723993	Os07g06950	1116 aa protein	unnamed protein product [Oryza sativa Japonica Group]
51	Accession: CAW79175.1 GI: 219894299	Os07g06950	1116 aa protein	unnamed protein product [Oryza sativa Japonica Group]
52	Accession: EEC82622.1 GI: 218200195	Os07g46660	737 aa protein	hypothetical protein OsI_27205 [Oryza sativa Indica Group]
53	Accession: EEE67751.1 GI: 222637619	Os07g46660	738 aa protein	hypothetical protein OsJ_25454 [Oryza sativa Japonica Group]
54	Accession: BAF22450.1 GI: 113612072	Os07g46660	489 aa protein	Os07g0661300, partial [Oryza sativa Japonica Group]

55	Accession: NP_001060536.1 GI: 115473875	Os07g46660	489 aa protein	Os07g0661300, partial [Oryza sativa Japonica Group]
56	Accession: BAC83574.1 GI: 34395185	Os07g46660	669 aa protein	putative ubiquitin-specific protease [Oryza sativa Japonica Group]
57	Accession: EAZ07363.1 GI: 125561915	Os08g37350	978 aa protein	hypothetical protein OsI_29613 [Oryza sativa Indica Group]
58	Accession: BAF24215.1 GI: 113624270	Os08g41530	959 aa protein	Os08g0527100 [Oryza sativa Japonica Group]
59	Accession: EAZ07724.1 GI: 125562276	Os08g41530	959 aa protein	hypothetical protein OsI_29979 [Oryza sativa Indica Group]
60	Accession: EAZ43413.1 GI: 125604088	Os08g41530	959 aa protein	hypothetical protein OsJ_28018 [Oryza sativa Japonica Group]
61	Accession: NP_001062301.1 GI: 115477411	Os08g41530	959 aa protein	Os08g0527100 [Oryza sativa Japonica Group]
62	Accession: BAG99642.1 GI: 215767414	Os08g41530	959 aa protein	unnamed protein product [Oryza sativa Japonica Group]
63	Accession: BAD08752.1 GI: 42407638	Os08g41530	846 aa protein	ubiquitin-specific protease-like [Oryza sativa Japonica Group]
64	Accession: EAZ07725.1 GI: 125562277	Os08g41540	869 aa protein	hypothetical protein OsI_29980 [Oryza sativa Indica Group]
65	Accession: EAZ43414.1 GI: 125604089	Os08g41540	869 aa protein	hypothetical protein OsJ_28019 [Oryza sativa Japonica Group]
66	Accession: BAD08753.1 GI: 42407639	Os08g41540	869 aa protein	ubiquitin-specific protease-like [Oryza sativa Japonica Group]
67	Accession: BAH00609.1 GI: 215768380	Os08g41550	527 aa protein	unnamed protein product [Oryza sativa Japonica Group]
68	Accession: BAF24218.1 GI: 113624273	Os08g41560	762 aa protein	Os08g0527400 [Oryza sativa Japonica Group]
69	Accession: NP_001062304.1 GI: 115477417	Os08g41560	762 aa protein	Os08g0527400 [Oryza sativa Japonica Group]
70	Accession: BAD08755.1 GI: 42407641	Os08g41560	817 aa protein	putative ubiquitin-specific protease [Oryza sativa Japonica Group]
71	Accession: BAG95418.1 GI: 215717055	Os08g41580	431 aa protein	unnamed protein product [Oryza sativa Japonica Group]
72	Accession: EAZ07731.1 GI: 125562283	Os08g41610	794 aa protein	hypothetical protein OsI_29986 [Oryza sativa Indica Group]
73	Accession: EAZ43420.1 GI: 125604095	Os08g41610	794 aa protein	hypothetical protein OsJ_28025 [Oryza sativa Japonica Group]

74	Accession: BAF24221.1 GI: 113624276	Os08g41610	390 aa protein	Os08g0527800 [Oryza sativa Japonica Group]
75	Accession: NP_001062307.1 GI: 115477423	Os08g41610	390 aa protein	Os08g0527800 [Oryza sativa Japonica Group]
76	Accession: BAG88679.1 GI: 215693297	Os08g41610	390 aa protein	unnamed protein product [Oryza sativa Japonica Group]
77	Accession: BAF24217.2 GI: 255678591	Os08g41610	82 aa protein	Os08g0527300, partial [Oryza sativa Japonica Group]
78	Accession: NP_001062303.2 GI: 297608871	Os08g41610	82 aa protein	Os08g0527300, partial [Oryza sativa Japonica Group]
79	Accession: BAD08758.1 GI: 42407644	Os08g41610	390 aa protein	ubiquitin-specific protease-like [Oryza sativa Japonica Group]
80	Accession: BAG95789.1 GI: 215736860	Os08g41620	750 aa protein	unnamed protein product [Oryza sativa Japonica Group]
81	Accession: BAF24224.1 GI: 113624279	Os08g41630	946 aa protein	Os08g0528100 [Oryza sativa Japonica Group]
82	Accession: EAZ07728.1 GI: 125562280	Os08g41630	838 aa protein	hypothetical protein OsI_29983 [Oryza sativa Indica Group]
83	Accession: EEE69038.1 GI: 222640906	Os08g41630	862 aa protein	hypothetical protein OsJ_28028 [Oryza sativa Japonica Group]
84	Accession: NP_001062310.1 GI: 115477429	Os08g41630	946 aa protein	Os08g0528100 [Oryza sativa Japonica Group]
85	Accession: BAD09096.1 GI: 42407958	Os08g41630	902 aa protein	ubiquitin-specific protease-like [Oryza sativa Japonica Group]
86	Accession: EEE69864.1 GI: 222641732	Os09g28940	940 aa protein	hypothetical protein OsJ_29670 [Oryza sativa Japonica Group]
87	Accession: BAD33960.1 GI: 50725209	Os09g28940	940 aa protein	putative ubiquitin-specific protease [Oryza sativa Japonica Group]
88	Accession: BAF25513.1 GI: 113631832	Os09g32740	1055 aa protein	Os09g0505100 [Oryza sativa Japonica Group]
89	Accession: EAZ09666.1 GI: 125564286	Os09g32740	1058 aa protein	hypothetical protein OsI_31949 [Oryza sativa Indica Group]
90	Accession: EAZ45285.1 GI: 125606249	Os09g32740	1055 aa protein	hypothetical protein OsJ_29927 [Oryza sativa Japonica Group]
91	Accession: NP_001063599.1 GI: 115480011	Os09g32740	1055 aa protein	Os09g0505100 [Oryza sativa Japonica Group]
92	Accession: BAG90503.1 GI: 215695312	Os09g32740	1055 aa protein	unnamed protein product [Oryza sativa Japonica Group]

93	Accession: EEC84994.1 GI: 218202567	Os09g37580	1173 aa protein	hypothetical protein OsI_32276 [Oryza sativa Indica Group]
94	Accession: EAZ45567.1 GI: 125606531	Os09g37580	1243 aa protein	hypothetical protein OsJ_30228 [Oryza sativa Japonica Group]
95	Accession: AAN04210.1 GI: 22655793	Os10g07270	890 aa protein	Putative ubiquitin carboxyl terminal hydrolase [Oryza sativa Japonica Group]
96	Accession: ABB46808.1 GI: 78707833	Os10g07270	931 aa protein	Ubiquitin carboxyl-terminal hydrolase family protein, expressed [Oryza sativa Japonica Group]
97	Accession: BAF26118.1 GI: 113638813	Os10g07270	931 aa protein	Os10g0160000 [Oryza sativa Japonica Group]
98	Accession: NP_001064204.1 GI: 115481222	Os10g07270	931 aa protein	Os10g0160000 [Oryza sativa Japonica Group]
99	Accession: BAG99462.1 GI: 215767234	Os10g07270	931 aa protein	unnamed protein product [Oryza sativa Japonica Group]
100	Accession: EEE50603.1 GI: 222612471	Os10g07270	931 aa protein	hypothetical protein OsJ_30790 [Oryza sativa Japonica Group]
101	Accession: EEC66592.1 GI: 218184165	Os10g07270	788 aa protein	hypothetical protein OsI_32808 [Oryza sativa Indica Group]
102	Accession: ABA93659.2 GI: 108864372	Os11g28360	382 aa protein	Ubiquitin carboxyl-terminal hydrolase 5, putative, expressed [Oryza sativa Japonica Group]
103	Accession: EEC68143.1 GI: 218185716	Os11g28360	918 aa protein	hypothetical protein OsI_36069 [Oryza sativa Indica Group]
104	Accession: EEE52082.1 GI: 222615950	Os11g28360	918 aa protein	hypothetical protein OsJ_33861 [Oryza sativa Japonica Group]
105	Accession: BAF25325.2 GI: 255678959	Os11g28360	918 aa protein	Os09g0464400 [Oryza sativa Japonica Group]
106	Accession: BAF28246.1 GI: 113645105	Os11g28360	382 aa protein	Os11g0473200 [Oryza sativa Japonica Group]
107	Accession: NP_001063411.2 GI: 297609602	Os11g28360	918 aa protein	Os09g0464400 [Oryza sativa Japonica Group]
108	Accession: NP_001067883.1 GI: 115485479	Os11g28360	382 aa protein	Os11g0473200 [Oryza sativa Japonica Group]
109	Accession: ABA94210.1 GI: 77551413	Os11g34270	500 aa protein	Ubiquitin carboxyl-terminal hydrolase family protein, expressed [Oryza sativa Japonica Group]
110	Accession: BAF28421.1 GI: 113645280	Os11g34270	500 aa protein	Os11g0545300 [Oryza sativa Japonica Group]

111	Accession: EEE52245.1 GI: 222616113	Os11g34270	631 aa protein	hypothetical protein OsJ_34188 [Oryza sativa Japonica Group]
112	Accession: NP_001068058.1 GI: 115485829	Os11g34270	500 aa protein	Os11g0545300 [Oryza sativa Japonica Group]
113	Accession: BAG89560.1 GI: 215694567	Os11g34270	500 aa protein	unnamed protein product [Oryza sativa Japonica Group]
114	Accession: ACZ52154.1 GI: 269914813	Os11g34270	714 aa protein	topoisomerase 6 subunit A-like protein [Oryza sativa Japonica Group]
115	Accession: ABA94399.1 GI: 77551602	Os11g36470	1451 aa protein	Ubiquitin carboxyl-terminal hydrolase family protein, expressed [Oryza sativa Japonica Group]
116	Accession: EEC68463.1 GI: 218186036	Os11g38630	388 aa protein	hypothetical protein OsI_36689 [Oryza sativa Indica Group]
117	Accession: EAY81516.1 GI: 125534968	Os11g38630	961 aa protein	hypothetical protein OsI_36685 [Oryza sativa Indica Group]
118	Accession: EAZ18903.1 GI: 125577681	Os11g38630	1017 aa protein	hypothetical protein OsJ_34443 [Oryza sativa Japonica Group]
119	Accession: ABA94711.1 GI: 77551914	Os11g38630	924 aa protein	hypothetical protein LOC_Os11g38630 [Oryza sativa Japonica Group]
120	Accession: EAY81679.1 GI: 125535131	Os11g40450	1307 aa protein	hypothetical protein OsI_36850 [Oryza sativa Indica Group]
121	Accession: EEE52481.1 GI: 222616349	Os11g40450	1609 aa protein	hypothetical protein OsJ_34660 [Oryza sativa Japonica Group]
122	Accession: ABA94767.2 GI: 108864585	Os11g40450	2446 aa protein	retrotransposon protein, putative, unclassified [Oryza sativa Japonica Group]
123	Accession: BAH95430.1 GI: 255680346	Os11g44540	119 aa protein	Os11g0667400 [Oryza sativa Japonica Group]
124	Accession: NP_001176702.1 GI: 297728677	Os11g44540	119 aa protein	Os11g0667400 [Oryza sativa Japonica Group]
125	Accession: Q0INW1.2 GI: 134035354	Os12g18760	640 aa protein	RecName: Full=Probable Ufm1-specific protease; Short=UfSP [Oryza sativa Japonica Group]
126	Accession: EEC69300.1 GI: 218186873	Os12g30540	1076 aa protein	hypothetical protein OsI_38365 [Oryza sativa Indica Group]
127	Accession: EAY81382.1 GI: 125534834	Os12g30540	1148 aa protein	hypothetical protein OsI_36553 [Oryza sativa Indica Group]
128	Accession: EEE53227.1 GI: 222617095	Os12g30540	1077 aa protein	hypothetical protein OsJ_36127 [Oryza sativa Japonica Group]

129	Accession: EEE52311.1 GI: 222616179	Os12g30540	1142 aa protein	hypothetical protein OsJ_34325 [Oryza sativa Japonica Group]
130	Accession: ABA98280.2 GI: 108862691	Os12g30540	1125 aa protein	ubiquitin-specific protease 12, putative, expressed [Oryza sativa Japonica Group]
131	Accession: ABA99902.2 GI: 108862978	Os12g42600	801 aa protein	Ubiquitin carboxyl-terminal hydrolase family protein, expressed [Oryza sativa Japonica Group]
132	Accession: EEC69695.1 GI: 218187268	Os12g42600	868 aa protein	hypothetical protein OsI_39158 [Oryza sativa Indica Group]
133	Accession: EEE53628.1 GI: 222617496	Os12g42600	882 aa protein	hypothetical protein OsJ_36905 [Oryza sativa Japonica Group]
134	Accession: BAF30317.2 GI: 255670491	Os12g42600	864 aa protein	Os12g0621000 [Oryza sativa Japonica Group]
135	Accession: NP_001067298.2 GI: 297613547	1Os2g42600	864 aa protein	Os12g0621000 [Oryza sativa Japonica Group]

Table S2 Rice OsUCH and OsUBP genes.

OsUCHs	*OsUBPs*		
Os02g08370	Os01g08200	Os06g08530	Os09g24250
Os02g43760	Os01g36930	Os06g44380	Os09g28940
Os02g57630	Os01g48600	Os07g06610	Os09g32740
Os04g46190	Os01g56490	Os07g06950	Os09g37580
Os04g57190	Os02g14730	Os07g46660	Os10g07270
	Os02g36400	Os08g37350	Os11g28360
	Os02g46650	Os08g41530	Os11g28365
	Os02g55180	Os08g41540	Os11g34270
	Os03g06950	Os08g41550	Os11g34690
	Os03g09080	Os08g41560	Os11g36470
	Os03g09260	Os08g41580	Os11g40450
	Os04g34984	Os08g41610	Os11g44540
	Os04g37950	Os08g41620	Os12g30540
	Os04g55360	Os08g41630	Os12g42600
	Os05g43480	Os09g17480	

Table S3 The *UCH* and *UBP* genes used for the generation of the phylogenetic tree.

	Uniprot	gene	NCBI	*tair（arabidopsis）*
ath: Arabidopsis thaliana	ath|O04482	UCH2	AY114549.1	At1g65650
	ath|O23592	UCH3	BT005488.1	At4g17510
	ath|Q8GWE1	UCH3	BT005488.1	At4g17510
	ath|Q945M7	UCH2	AY114549.1	At1g65650
	ath|Q9FFF2	UCH1	BT005312.1	At5g16310
	ath|Q9SHY9	UCH2	AY114549.1	At1g65650
hum:human	hum|P09936	UCH L1	AH007277.2	
	hum|P15374	UCH L3	M30496	
	hum|Q9Y5K5	UCH L5	BT006790	
moa:Monopterus albus	moa|B5KNK6	UCH L1	EU095955	
mou:mouse	mou|P58321	UCH L4	AB035420	
	mou|Q9JKB1	UCH L3	AF247358	
	mou|Q9R0P9	UCH L1	AB025313	
	mou|Q9WUP7	UCH L5	AK011117	
osm: Oryza sativa	osm2g08370		AK067359	Os02g08370
	osm2g43760		AK066320	Os02g43760
	osm2g57630		AK058380	Os02g57630
	osm4g46190		AK059677	Os04g46190
	osm4g57190		AK103595	Os04g57190
pop: Populus trichocarpa	pop|B9GYN0		CM000339	
	pop|B9H3Y8		CM000340	
	pop|B9IJQ6		CM000353	
ppa: Physcomitrella patens	ppa|A9SZ40		DS545029	
	ppa|A9RL89		DS544904	
rat：Rattus norvegicus	rat|Q00981	UCH L1	BC060573	
	rat|Q91Y78	UCH L3	AB043959	
Sbi: Sorghum bicolor	sbi|C5WRS1		CM000760	
	sbi|C5XS03		CM000763	
	sbi|C5YA10		deleted	
	sbi|C5YDE1		CM000765	
	sbi|C5YMV1		CM000766	
	sce|A6ZQ55		AAFW02000040	

Sce: Saccharomyces cerevisiae	sce|B3LQI4		CH408050
	sce|B5VLP6		ABSV01001378
	sce|C7GMA8		ACFL01000054
	sce|C8ZBP6		FN393076
	sce|E7KEI8		ADVS01000033
	sce|E7KQL9		ADVV01000053
	sce|E7LWH0		ADXC01000046
	sce|E7NJH5		AEEZ01000057
	sce|E7Q5R4		AEHH01000043
	sce|E7QGV6		AEJS01000043
	sce|P35127		Z49599
Smo: Selaginella moellendorffii	smo|D8QRL6		GL377566
	smo|D8REF4		GL377577
	smo|D8RHZ4		GL377580
	smo|D8RJ95		GL377581
Spo: Schizosaccharomyces pombe	spo|Q10171	UCH1	CU329670
	spo|Q9UUB6	UCH2	CU329671
zm: Zea mays	zm|B4F819		BT033257
	zm|B4F8D8		BT033376
	zm|B4FN14		BT038502
	zm|B6SI96		EU952461
	zm|B6T8R6		EU961381
	zm|B6TLS0		EU965935
	zm|B6U4S6		EU972241
	zm|B8A3F6		BT056098
	zm|C0HHI4		BT061790
	zm|C0HI50		BT062006
	zm|C0P3H1		BT062840

Table S4-2 UBP genes for Phylogenetic tree.

	Uniprot	gene	NCBI	*tair（arabidopsis）*
ath: Arabidopsis thaliana	ath|O22207	UBP5	AF048705	At2g40930
	ath|O24454	UBP3	U76845	At4g39910
	ath|Q67XW5	UBP18	AL031004	At4g31670
	ath|Q84WC6	UBP7	AF302661	At3g21280
	ath|Q84WU2	UBP13	AC016795	At3g11910

	ath|Q8L6Y1	UBP14	AF302664	At3g20630
	ath|Q8LAM0	UBP4	U76846	At2g22310
	ath|Q8W4N3	UBP2	AF302659	At1g04860
	ath|Q93Y01	UBP9	AF118222	At4g10590
	ath|Q949Y0	UBP6	AF302660	At1g51710
	ath|Q9C585	UBP8	AL589883	At5g22030
	ath|Q9FIQ1	UBP21	AF302669	At5g46740
	ath|Q9FKP5	UBP17	AF302667	At5g65450
	ath|Q9FPS0	UBP27	AF302675	At4g39370
	ath|Q9FPS2	UBP25	AF302673	At3g14400
	ath|Q9FPS3	UBP24	AF302672	At4g30890
	ath|Q9FPS4	UBP23	AF302671	At5g57990
	ath|Q9FPS7	UBP20	AF302668	At4g17895
	ath|Q9FPS9	UBP15	AF302665	At1g17110
	ath|Q9FPT1	UBP12	F302663	At5g06600
	ath|Q9FPT5	UBP1	AC003974	At2g32780
	ath|Q9LEW0	UBP22	AF302670	At5g10790
	ath|Q9MAQ3	UBP11	AC006424	At1g32850
	ath|Q9SB51	UBP16	AF302666	At4g24560
	ath|Q9SCJ9	UBP26	Q9SCJ9	At4g24560
	ath|Q9SJA1	UBP19	AC006954	At2g24640
	ath|Q9ZSB5	UBP10	AF118222	At4g10570
hum:human	hum|A6NNY8	USP27X	AF238380	
	hum|O75317	USP12	AF022789	
	hum|O75604	USP2	AF079564	
	hum|O94782	USP1	AB014458	
	hum|O94966	USP19	AB020698	
	hum|P35125	USP6	X63546	
	hum|P40818	USP8	D29956	
	hum|P45974	USP5	X91349	
	hum|P51784	USP11	AL096791	
	hum|P54578	USP14	U30888	
	hum|P62068	USP46	GU455414	
	hum|Q0WX57	USP17	AF544011	
	hum|Q13107	USP4	U20657	
	hum|Q14694	USP10	D80012	
	hum|Q70CQ1	USP49	AJ586139	

	hum|Q70CQ2	USP34	AJ586138
	hum|Q70CQ3	USP30	AJ586136
	hum|Q70CQ4	USP31	AJ586135
	hum|Q70EK9	USP51	AJ583823
	hum|Q70EL2	USP45	AJ583819
	hum|Q70EL4	USP43	AJ583817
	hum|Q86T82	USP37	AL832645
	hum|Q86UV5	USP48	AF502942
	hum|Q8NB14	USP38	AK057992
	hum|Q8NFA0	USP32	AF533230
	hum|Q8TEY7	USP33	AF383172
	hum|Q92995	USP13	U75362
	hum|Q93009	USP7	Z72499
	hum|Q96K76	USP47	AK000734
	hum|Q96RU2	USP28	AF266283
	hum|Q9BXU7	USP26	AF285593
	hum|Q9H0E7	USP44	AL136825
	hum|Q9H9J4	USP42	AJ601395
	hum|Q9HBJ7	USP29	AF229438
	hum|Q9NVE5	USP40	AJ583821
	hum|Q9P275	USP36	AB040886
	hum|Q9P2H5	USP35	AJ586137
	hum|Q9UHP3	USP25	AF170562
	hum|Q9UK80	USP21	AF177758
	hum|Q9UPT9	USP22	AB028986
	hum|Q9UPU5	USP24	AC091609
	hum|Q9Y2K6	USP20	AY074877
	hum|Q9Y4E8	USP15	AF106069
	hum|Q9Y5T5	USP16	AF126736
	hum|Q9Y6I4	USP3	AF073344
mou:mouse	mou|B1AQJ2	USP36	AL591404
	mou|B1AY13	USP24	AK045043
	mou|B2RQC2	USP42	CH466529
	mou|O88623	USP2	AY255637
	mou|P35123	USP4	AF026469
	mou|P52479	USP10	D84096
	mou|P56399	USP5	AC002397

	mou|P57080	USP25	AF170563
	mou|P62069	USP46	AK145298
	mou|Q3UJD6	USP19	AK122396
	mou|Q3UN04	USP30	AK078164
	mou|Q3V0C5	USP48	AK220190
	mou|Q5DU02	USP22	AK084022
	mou|Q5I043	USP28	BC066033
	mou|Q6A4J8	USP7	AF548565
	mou|Q6P9L4	USP49	BC060712
	mou|Q6ZQ93	USP34	AL672049
	mou|Q80U87	USP8	AB045709
	mou|Q8BJQ2	USP1	AK080882
	mou|Q8BUM9	USP43	AL732570
	mou|Q8BW70	USP38	AK049287
	mou|Q8BWR4	USP40	AK050239
	mou|Q8BY87	USP47	AK030909
	mou|Q8C0R0	USP37	AK030013
	mou|Q8C6M1	USP20	AF449715
	mou|Q8CEG8	USP27	AF229643
	mou|Q8K387	USP45	AK036903
	mou|Q8R5H1	USP15	AF468037
	mou|Q8R5K2	USP33	AF383174
	mou|Q91W36	USP3	AK031141
	mou|Q99K46	USP11	AK166523
	mou|Q99LG0	USP16	AK160038
	mou|Q99MX1	USP26	AF285570
	mou|Q9D9M2	USP12	AK006739
	mou|Q9ES63	USP29	AF229257
	mou|Q9JMA1	USP14	AB034633
	mou|Q9QZL6	USP21	AF177759
osm: Oryza sativa	os01g08200		AK103230
	os01g36930		AK066233.1
	os01g48600		AP003335.4
	os01g56490		AK242694.1
	os02g14730		AK121968.1
	os02g36400		AK287848.1
	os02g46650		AK069300.1

os02g55180	AK120097.1
os03g06950	AK062304.1
os03g09080	AK069100.1
os03g09260	AK110648.1
os04g34984	AK070730.1
os04g37950	AK068069.1
os04g55360	AK100969.1
os05g43480	AK120186.1
os06g08530	AK102933
os06g44380	AK067618.1
os07g06610	AP003847.3
os07g06950	AK241926.1
os07g46660	AK060418
os08g37350	AK099704.1
os08g41530	AK119411.1
os08g41540	AP003857.3
os08g41550	AK121686.1
os08g41560	AK119389.1
os08g41580	AK102160.1
os08g41610	AK063392.1
os08g41620	AP003857.3
os08g41630	AP004015.3
os09g17480	AP005881.3
os09g24250	AK070971.1
os09g28940	AP005676.3
os09g32740	AK067621.1
os09g37580	AP005862.2
os10g07270	AK111889.1
os11g28360	AK106730.1
os11g28365	AK330261.1
os11g34270	AK065551.1
os11g34690	AK287786.1
os11g36470	AK059481.1
os11g40450	AF161269.1
os11g44540	NM_001065471.1
os12g30540	AK059573
os12g42600	NM_001073830.1

php: Physcomitrella patens	php|A9SG48		DS544964
	php|A9U459		DS545372
pop: Populus trichocarpa	pop|B9GFW8		CM000337
	pop|B9I019		CM000347
	pop|B9IJH8		CM000353
	pop|B9IMS0		CM000354
	pop|B9N564		CM000337
rat：Rattus norvegicus	rat|B2GUX4	USP21	BC166443
	rat|B2GUZ1	USP4	BC166460
	rat|D3ZJ96	USP28	AB240643
	rat|D3ZU96	USP42	CH474012
	rat|Q2KJ09	USP16	CH473989
	rat|Q3KR59	USP10	BC105892
	rat|Q4VSI4	USP7	AY641530
	rat|Q569C3	USP1	BC092574
	rat|Q5U349	USP2	AF202453
	rat|Q6J1Y9	USP19	AY605065
	rat|Q76LT8	USP48	AB073880
Sbi: Sorghum bicolor	sbi|C5X4P2		CM000761
	sbi|C5XMS9		CM000762
	sbi|C5YHM5		CM000766
	sbi|C5YI45		CM000766
Sce: Saccharomyces cerevisiae	sce|A6ZL02	UBP14	AAFW02000011
	sce|B3LGK1	UBP4	CH408043
	sce|P25037	UBP1	M63484
	sce|P32571	UBP4	U02518
	sce|P36026	UBP11	Z28323
	sce|P38187	UBP13	Z35828
	sce|P38237	UBP14	Z35927
	sce|P39538	UBP12	X77688
	sce|P39944	UBP5	U10082
	sce|P39967	UBP9	U18839
	sce|P40453	UBP7	Z38059
	sce|P43593	UBP6	D50617
	sce|P50101	UBP15	Z49212
	sce|P50102	UBP8	Z49939
	sce|P53874	UBP10	Z71462

	sce|Q01476	UBP2	M94916
	sce|Q01477	UBP3	M94917
Smo: Selaginella moellendorffii	smo|D8R6K7		GL377572
	smo|D8RS26		GL377588
	smo|D8S4D5		GL377601
	smo|D8SJH1		GL377623
Spo: Schizosaccharomyces pombe	spo|Q11119	UBP14	CU329671
	spo|Q92353	UBP6	CU329670
	spo|Q9UTT1	UBP21	AF187961
	spo|Q9UUD6	UBP11	CU329671
zm: Zea mays	zm|B6SW43	UBP25	EU956958
	zm|C0PCY1		BT066150

Table S4 The domain locations of rice OsUBPs.

oups	OsUBPs	Full length	Domain	Domain sites
Group1	Os09g32740	1055aa	Znf-UBP	91-191
			Peptidase C19	225-1054
	Os08g41530	959aa	Znf-UBP	128-200
			Znf-UBP	237-309
			Peptidase C19	340-958
	Os08g41610	794aa	Znf-UBP	78-141
			Peptidase C19	193-793
	Os08g41620	750aa	Znf-UBP	133-195
			Peptidase C19	244-749
	Os08g41630	946aa	Znf-UBP	126-188
			Znf-UBP	274-334
			Peptidase C19	364-945
	Os08g41550	914aa	Znf-UBP	74-146
			Znf-UBP	203-274
			Peptidase C19	305-913
	Os08g41560	817aa	Znf-UBP	89-161
			Peptidase C19	205-816
	Os08g41540	869aa	Znf-UBP	82-158
			Znf-UBP	200-272
			Peptidase C19	303-868
	Os11g40450	2446aa	Retrotrans gag	99-185
			RVT_2	632-754
			RVT_2	756-841
			Peptidase C19	2050-2425
	Os08g41580	835aa	Znf-UBP	217-289
			Peptidase C19	311-834
Group2	Os03g09080	366aa	Peptidase C19	23-363
	Os04g37950	367aa	Peptidase C19	25-364
	Os02g36400	415aa	Peptidase C19	72-412
Group3	Os01g36930	474aa	Ubiquitin	2-73
			Peptidase C19	104-473
Group4	Os10g07270	931aa	DUSP	18-138
			Peptidase C19	311-909
	Os11g28360	918aa	DUSP	16-141
			Peptidase C19	323-897
	Os12g42600	801aa	Peptidase C19	271-781
	Os11g44540	109aa	Peptidase C19	73-109

	Os01g48600	97aa	Peptidase C19	1-97
	Os07g06610	951aa	Peptidase C19	262-862
	Os09g17480	423aa	Peptidase C19	109-154
	Os11g28365	327aa	Peptidase C19	1-306
Group5	Os01g56490	1108aa	MATH	50-173
			Peptidase C19	193-518
	Os12g30540	1125aa	MATH	62-187
			Peptidase C19	207-532
	Os11g36470	1451aa	MATH	54-179
			Peptidase C19	199-503
			Tify	1279-1314
			CCT	1341-1383
			Znf-GATA	1400-1435
	Os07g06950	1116aa	MATH	77-202
			Peptidase C19	217-528
Group6	Os01g08200	794aa	Znf-UBP	181-255
			UBA	610-648
			UBA	671-708
			Peptidase C19	311-794
Group7	Os09g28940	940aa	Znf-MYND	118-155
			Peptidase C19	382-701
	Os08g37350	897aa	Znf-MYND	42-79
			Peptidase C19	369-676
	Os06g44380	1069aa	Znf-MYND	105-142
			Peptidase C19	204-512
	Os02g14730	907aa	Znf-MYND	88-125
			Peptidase C19	409-716
	Os02g55180	1185aa	Peptidase C19	86-391
	Os06g08530	842aa	Peptidase C19	2-307
	Os05g43480	644aa	Peptidase C19	23-345
Group8	Os02g46650	672aa	Peptidase C19	1-445
Group9	Os04g55360	562aa	Znf-UBP	39-112
			Peptidase C19	182-550
	Os09g24250	554aa	Znf-UBP	124-185
			Peptidase C19	227-554
Group10	Os07g46660	669aa	Peptidase C19	316-669
Group11	Os03g09260	1079aa	Peptidase C19	106-447
			DUSP	495-598
			DUSP	613-716
			DUSP	738-862
			ubiquitin	966-1029

Group12	Os04g34984	538aa	Peptidase C19	64-538
Group13	Os11g34270	500aa	Peptidase C19	1-183
Group14	Os09g37580	1243aa	TPR-like	29-160
			DUF629	348-422
			Peptidase C19	920-1239
	Os03g06950	1579aa	DUF627	45-155
			DUF629	320-811
			Peptidase C19	1273-1579
	Os11g34690	898aa	TPR-like	37-122
			DUF629	189-303
			Peptidase C19	582-853

Table S5 The OUT and JAMM family members.

Otubain Protease（OTU）

	Gene ID（NCBI）	RAPDB_Locus	MSU_Locus	Gene description
1	LOC4337352	Os04g0670400	Os04g57480	OTU domain-containing protein 3
2	LOC4335795	Os04g0414100	Os04g33780	OTU domain-containing protein 5
3	LOC4331162	Os02g0819500	Os02g57410	OTU domain-containing protein 5-A
4	LOC4337022	Os04g0619500	Os04g52850	OTU domain-containing protein 6B
5	LOC4325039	Os01g0900900	Os01g67490	OTU domain-containing protein At3g57810
6	LOC4332354	Os03g0266000	Os03g15930	OTU domain-containing protein At3g57810
7	LOC4347429	Os09g0487700	Os09g31280	OTU domain-containing protein At3g57810
8	LOC4328430	Os02g0168600	Os02g07210	OTU domain-containing protein DDB_G0284757
9	LOC4335733	Os04g0402300	Os04g32970	OTU domain-containing protein DDB_G0284757
10	LOC4341800	Os06g0669800	Os06g45850	OTU domain-containing protein DDB_G0284757
11	LOC4328405	Os02g0164800	Os02g06890	ubiquitin thioesterase OTU1
12	LOC107276635	Os02g0521500	Os02g32180	ubiquitin thioesterase otubain-like
13	LOC4329466	Os02g0513800	Os02g30974	uncharacterized LOC4329466
14	LOC4329505	Os02g0522700	Os02g32280	uncharacterized LOC4329505
15	LOC4329506	Os02g0522800	Os02g32290	uncharacterized LOC4329506
16	LOC4333353	Os03g0589300	Os03g39230	uncharacterized LOC4333353
17	LOC4334861	Os03g0859800	Os03g64219	uncharacterized LOC4334861
18	LOC4345979	Os08g0506000	Os08g39560	uncharacterized LOC4345979
19	LOC9268497	Os02g0517600	Os02g31830	uncharacterized LOC9268497
20	LOC102702173			OTU domain-containing protein At3g57810
21	LOC102707990			OTU domain-containing protein At3g57810-like
22	LOC102710685			ubiquitin thioesterase otubain-like
23	LOC102717235			uncharacterized LOC102717235

24	LOC102709836			OTU domain-containing protein 3
25	LOC102703574			OTU domain-containing protein 5
26	LOC102722185			OTU domain-containing protein 5-A-like
27	LOC102707023			OTU domain-containing protein 6B
28	LOC102709765			OTU domain-containing protein At3g57810
29	LOC102720014			OTU domain-containing protein At3g57810-like
30	LOC102718641			OTU domain-containing protein DDB_G0284757
31	LOC102707594			OTU domain-containing protein DDB_G0284757-like
32	LOC102700681			OTU domain-containing protein DDB_G0284757-like
33	LOC102720672			ubiquitin thioesterase OTU1
34	LOC107303486			ubiquitin thioesterase OTUB1-like
35	LOC107303605			ubiquitin thioesterase otubain-like
36	LOC102703099			ubiquitin thioesterase otubain-like
37	LOC102699379			uncharacterized LOC102699379
38	LOC102702823			uncharacterized LOC102702823
39	LOC102713350			uncharacterized LOC102713350

JAB1/MPN/Mov34 Metalloenzyme（JAMM）

	Gene symbol/Gene ID（NCBI）	RAPDB_Locus	MSU_Locus	Gene description
1	LOC102705344			26S proteasome non-ATPase regulatory subunit 14 homolog
2	LOC102704461			26S proteasome non-ATPase regulatory subunit 14 homolog
3	LOC102717105			AMSH-like ubiquitin thioesterase 2
4	LOC102712024			AMSH-like ubiquitin thioesterase 3
5	LOC102705380			AMSH-like ubiquitin thioesterase 3
6	LOC102712302			AMSH-like ubiquitin thioesterase 3
7	LOC102715012			COP9 signalosome complex subunit 5b-like
8	LOC4341987	Os06g0703600	Os06g49020	26S proteasome non-ATPase regulatory subunit 14 homolog
9	LOC4338586	Os05g0371200	Os05g30800	26S proteasome non-ATPase regulatory subunit 14 homolog
10	LOC4341987	Os06g0703600	Os06g49020	26S proteasome non-ATPase regulatory subunit 14 homolog

11	LOC4324379	Os01g0267200	Os01g16190	26S proteasome non-ATPase regulatory subunit 14 homolog
12	LOC4338586	Os05g0371200	Os05g30800	26S proteasome non-ATPase regulatory subunit 14 homolog
13	LOC4327852	Os01g0661500	Os01g47262	AMSH-like ubiquitin thioesterase 2
14	LOC4324542	Os01g0499300	Os01g31470	AMSH-like ubiquitin thioesterase 3
15	LOC4326798	Os01g0338200	Os01g23640	AMSH-like ubiquitin thioesterase 3
16	LOC4337249	Os04g0654700	Os04g56070	COP9 signalosome complex subunit 5b

Table S6 Data used for kinetics calculation.

		m （g）	M (KD)	V_{max} (umol·mg^{-1}·min^{-1})	K_m (M)	K_{cat} (S^{-1})
Enzyme	OsUCH3	0.1	24.869			
Substrate	Ub-AMC					
	R1			5.00E-07	1.73E-07	
	R2			5.00E-07	1.84E-07	
	R3			5.00E-07	1.99E-07	
	Average			5.00E-07	1.85E-07	7.46
	SD			0.00E+00	1.30E-08	0.00
	NEDD8-AMC					
	R1			1.00E-06	1.46E-07	
	R2			1.14E-06	1.67E-07	
	R3			1.38E-06	2.05E-07	
	Average			1.17E-06	1.73E-07	17.22
	SD			1.94E-07	3.02E-08	2.89

R1-3: three independent replicates.

Table S7 Detection of enzyme activity.

Ub-AMC as substrate:

	protein	Inhibitor (2ml system)	substrate
pCold	pCold 50ug/ml		Ub-AMC
HsUCHL3	HsUCHL3 50ug/ml		Ub-AMC
OsUCH3	OsUCH3 50ug/ml		Ub-AMC
OsUCH3+Ub-VME	OsUCH3 50ug/ml	1ul 10Um Ub-VME	Ub-AMC
OsUCH3C96S	OsUCH3C96S 50ug/ml		Ub-AMC

NEDD8-AMC as substrate:

	protein	Inhibitor (2ml system)	substrate
pCold	pCold 50ug/ml		NEDD8-AMC
HsUCHL3	HsUCHL3 50ug/ml		NEDD8-AMC
OsUCH3	OsUCH3 50ug/ml		NEDD8-AMC
OsUCH3+Ub-VME	OsUCH3 50ug/ml	1ul 10Um Ub-VME	NEDD8-AMC
OsUCH3C96S	OsUCH3C96S 50ug/ml		NEDD8-AMC

Table S8 OsUCHs- and OsUBPs-family genes that are preferentially expressed in rice stamens.

Gene/group	locus	Stamen preferentially expression in microarray (Chen et al., 2015)	Stamen preferentially expression by RT-qPCR	Stamen preferentially expression by *in situ* hybridization	Enzymatic activity
OsUCHs	Os02g43760	+	+	+	+
	Os04g46190	+	+	+	+
	Os04g57190	+			+
OsUBPs/G1	Os08g41620			+	+
	Os08g41630			+	
	Os09g32740	+			+
OsUBPs/G2	Os04g37950	+			
OsUBPs/G3	Os01g36930	+			+
OsUBPs/G4	Os07g06610	+	+	+	+
	Os01g48600		+	+	
	Os10g07270	+			+
OsUBPs/G5	Os01g56490	+			+
OsUBPs/G6	Os01g08200	+	+		+
OsUBPs/G7	Os02g14730	+	+	+	
	Os06g08530	+			
	Os06g44380	+			
	Os08g37350	+			

OsUBPs/G9	Os09g24250	+	+
OsUBPs/G15	Os09g37580	+	+

Note: + is positive result.

Table S9 List of primers used for *In situ* hybridization, vector construction, RNA level detection and qRT-PCR.

Primers for in vitro enzyme activity detection vectors

gene	Primer name	Primers sequence（5'-3'）
Os02g43760	pCold 320-F	GGAATTCcatATGGGGAAGCGGTGG（NdeI）
（AK066320）	pCold 320-R	ACGCgtcgacTTACACAACTTTCGAAAGAGCC（SalI）
	320-C96S-F	CAACGCATCCGGAACAGTTGG
	320-C96S-R	CCAACTGTTCCGGATGCGTTG
Os02g08370	pCold 359-F1	CCGgaattcATGTCTTGGGCTGCAATCGAGAA（EcoRI）
（AK067359）	pCold 359-R	ACGCgtcgacTCACCTAGGATTAGCAGAGCTGTG（SalI）
	359C81S-F	AACAATGCATCTGCCACCCAAGC
	359C81S-R	GCTTGGGTGGCAGATGCATTGTT
Os02g57630	pCold 380-F	GGAATTCcatATGTCGTGGTGCACGATTGAGTCTG（NdeI）
（AK058380）	pCold 380-R	ACGCgtcgacTCATCTTGCACTCGTGCTTGTGCT（SalI）
	380C82S-F	CCCTAATGCATCTGCTACTCAAGC
	380C82S-R	GCTTGAGTAGCAGATGCATTAGGG
Os04g57190.1	pCold595.1-F	GGAATTCcatATGGCAGCGGTGCCCTCCG（NdeI）
（AK103595）	pCold595-R	ACGCgtcgacTTACTCTGCTTTTCTTGATAGCAC（SalI）
	595C107S-F	GAAATGCTTCTGGAACTATTGCGC
	595C107S-R	GCGCAATAGTTCCAGAAGCATTTC
Os04g46190	pCold 677-F	GGAATTCcatATGGGCAAGCGCTGGCTCC（NdeI）
（AK059677）	pCold 677-R	ACGCgtcgacTCACTGCTTTGAAAGAGCCATGA（SalI）
	677-C91S-F	GGTAATGCCTCTGGAACAGTTGG
	677-C91S-R	CCAACTGTTCCAGAGGCATTACC

Primers for in vivo enzyme activity detection vectors

gene	Primer name	Primers sequence（5'-3'）
AtUBQ10	AtUBQ10- BamHI	CGCggatccGATGCAGATTTTCGTTAAAACCCTAAC
	AtUBQ10-HindIII-R	CCCaagcttTTAGAAACCACCACGAAGACG
Os02g43760	320-EcoRV	gatatcGATGGGGAAGCGGTGG
（AK066320）	320-KpnI	CGGggtaccTTACACAACTTTCGAAAGAGCC
Os02g08370	359-EcoRV	atcGATGTCTTGGGCTGCAATCGAGAA
（AK067359）	359-KpnI	CGGggtaccTCACCTAGGATTAGCAGAGCTGTG
Os02g57630	380-EcoRV	atcGATGTCGTGGTGCACGATTGAGTCTG
（AK058380）	380-KpnI	CGGggtaccTCATCTTGCACTCGTGCTTGTGCT
Os04g57190.1	595-EcoRV	atcGATGGCAGCGGTGCCCTCCG
（AK103595）	595-KpnI	CGGggtaccTTACTCTGCTTTTCTTGATAGCAC

Os04g46190（AK059677）	677- EcoRV	gatatcGATGGGCAAGCGCTGGCTCC
	677-KpnI	CGGggtaccTCACTGCTTTGAAAGAGCCATGA
Os09g32740	32740-EcoRV	atcGATGGGGAAGAGGGTGAAGG
	32740-KpnI	CGGggtaccTTAGTCTCCCACCCTTTCGT
Os08g41620	41620-KpnI	GGggtaccATGGACGACGAGAAGAGGC
	41620-XhoI	CCctcgagTTAGCCTTCAATCCTCTCATAGAAAAG
Os08g41630	41630-NdeI	GGAATTCcatATGGCTGAATTCTCGTCAGCG
	41630-XhoI	CCctcgagTCAGCCTTCCATCCTCTCATAG
Os04g37950	37950-EcoRV	atcGATGGTCATGGGAGCCAGCGG
	37950-KpnI	CGGggtaccCTAACTCTTCCCACCAAGAC
Os01g36930	36930-EcoRV	atcGATGCCGACCGTAAGCGTGAAAT
	36930-KpnI	CGGggtaccTCAGATAACACGAGCTTTGTACAG
	36930C113S-F	CTTGGGGAATACATCTTACATGAATTCCACT
	36930C113S-R	AGTGGAATTCATGTAAGATGTATTCCCCAAG
Os10g07270	07270-KpnI	GGggtaccATGACGATACCGAGCGCCGA
	07270-XhoI	CCctcgagCTATGTGTCCAGCGAATCA
Os01g48600	48600-EcoRV	atcGATGAAGAAATTAGATCTGTGGAGGC
	48600-KpnI	CGGggtaccTCATTCTCGTGTGTAGAACAGC
Os07g06610	06610-KpnI-F	CGCTGACGTCggtaccATGGCCACCGCCGCCAC
	06610-KpnI-R	CTTTACCAGACTCGAGggtaccTCATTCTCGTCTGTAGAACAGCACA
Os01g56490	56490-KpnI-F	GATCGCTGACGTCggtaccATGACCATGGTGACCCCG
	56490-KpnI-R	TCTTTACCAGACTCGAGggtaccCTAGTTGAAAATCTTCACAGCCTTA
Os12g30540	30540-KpnI	GGggtaccATGACTATGATGACTCCTCC
	30540-XhoI	CCctcgagTCAATTATAAATTTTCACAG
Os07g06950	06950-EcoRV	atcGATGTATTTGGATGTGGCTGATT
	06950-KpnI	CGGggtaccCTAGTTATAGATCTTTACAGGTCTCT
Os01g08200	08200-KpnI	GGggtaccATGGATCTCCTCCGCTCGCA
	08200-XhoI	CCctcgagTTATATCCTTTGAAAGAAAT
Os06g08530.4	933-KpnI	GGggtaccATGCTAGACTTGAAGCCATT
	933-XhoI	CCctcgagTCATCTTCTAACATGGGCAT
Os07g46660.2	46660.2-EcoRV	atcGATGAGCGGCGGCGGCGGCAG
	46660.2-KpnI	CGGggtaccTTATAATTGTTTGTAGAACA
Os11g34270	551-NdeI	GGAATTCcatATGTTTGGTGGAAAATTCCGTCG
	551-XhoI	CCctcgagTTATAAGTATATCTGGAGCCCAT
Os09g37580	37580-KpnI	GGggtaccATGCGCAGAGGAGCATCCCAAG
	37580-XhoI	CCctcgagCTACTTGATGACCTCATAGACAAGGAC
AK101994	994-NdeI	GGAATTCcatATGGACTGGGACAGCGCCAC
	994-KpnI	GGggtaccCTAGACTGTGACTTCTGTGCCCGC

Primers for construction of OsUCH3 prokaryotic expression vector (detected by circular dichroism)

gene	Primer name	Primers sequence（5’-3’）
OsUCH3（AK066320）/	pET320-EcoRI	CGgaattcGATGGGGAAGCGGTGG
OsUCH3C96S	pET320-HindIII	CCCaagcttTTACACAACTTTCGAAAGAGCC

Primers for RT-qPCR

subfamily	name	gene	ID	5' RT Primer	3' RT Primer
Group1	G1-1	Os08g41530	AK119411.1	ATGTGCCCACTTCGTACGTT	AGGTAATGCTCACACCTCGC
	G1-2	Os08g41540	AP003857.3	TCCACTGCCATCAAGGAACA	TGAGAATCACGTCCTTCAGCAA
	G1-3	Os08g41550	AK121686.1	GCAGCGTGGAGACTTCTCAT	GATGGCACACGACAGCATTC
	G1-4	Os08g41560	AK119389.1	TGATCCTCAGGTTCCCGCTT	ACCAACTACCAAAGGCTCTGA
	G1-5	Os08g41580	AK102160.1	TCCTTGGTGACTTGCTGCAT	TAACGAAAGCGCACACAACG
	G1-6	Os08g41610	AK063392.1	AGTTGCTCCAAGGTTGCTCA	GCTCGCTTCGGCATGTTATC
	G1-7	Os08g41620	AP003857.3	TGGAGTCGGTCTCAAGTCCT	GAGAGAGGCAGCATTTGGGT
	G1-8	Os08g41630	AP004015.3	TGAGACAAAGTCGTCCGCAG	AGCCTCGCACTTGAGAACTT
	G1-9	Os09g32740	AK067621.1	ACAGTGCTGAAGACGAGGTG	AGTGCCATGGTTCAGAGAGC
	G1-10	Os11g40450	AF161269.1	AACATTCCCAGCCCCAACAA	TCAAGCCTGCAACGGAGTAG
Group2	G2-1	Os02g36400	AK287848.1	TAAAGAAGGCGCCACACGTA	AGCTTCAGCTCCAGGGGATA
	G2-2	Os03g09080	AK069100.1	TGTGTTCCTTTCCGTGAGCA	ACAGGTCAGCTAAGCACGTT
	G2-3	Os04g37950	AK068069.1	GCGAGCGCTACTTCGGCCTC	AAAGGTTCTCCTCCGCATCTCCAGG
Group3	G3	Os01g36930	AK066233.1	CTCGTGAGCAGGGTCCATTGTGC	GCAGTGCGACCCAGTGAAGGTG
Group4	G4-1	Os01g48600	AP003335.4	GTGGAGGCTACCGGAAGTT	GAAACCAACCAGCCTTGCTC
	G4-2	Os07g06610	AP003847.3	TACACCGCCAGCATCTACCA	TCTCGTCTGTAGAACAGCACA
	G4-3	Os09g17480	AP005881.3	ACCGCTTCAGTCATAGCAGG	GACTAATGCGGTGCTTGTGT
	G4-4	Os10g07270	AK111889.1	CGGCGGAATGGGTGGTGGTC	GCAGGAGCTGCTACCAACTCGC
	G4-5	Os11g28360	AK106730.1	CTGCGATCTTCGAATGGGGA	CCCAAGTTGAGTAGCCCTGT
	G4-6	Os11g28365	AK330261.1	TCAGTTTGTTCATCGCCGTG	GAACACCGTAGGGCTTCCAA
	G4-7	Os11g44540	NM_001065471.1	CAATGTGGTACTGCCCATGC	TTCCGGTAGCCTCCACAGAT
	G4-8	Os12g42600	NM_001073830.1	TCGCAGATGAGTACTGGGGA	ACAACCACCCCAGCTAGAGA

Group5	G5-1	Os01g56490	AK242694.1	AGAGGCCCACATGTTCACTG	CCCGAAGGCTTTGGATGTTG
	G5-2	Os07g06950	AK241926.1	GTGACCACAAGCGCCAATTT	AGCATTGGCGTGTAGAACGA
	G5-3	Os11g36470	AK059481.1	CCAACGCTATCAGATCAATGG TAT	ACTCTTCCTCACCCCCGTAT
	G5-4	Os12g30540	AK059573	ATCGCTGAGCATTTGCGGAT	TCCTCATCTCGGGCAACCTT
Group6	G6	Os01g08200	AK103230	ACCTCCACATCGTGCAGCGC	CGATGGCGAGTAGCGTGGGC
Group7	G7-1	Os02g14730	AK121968.1	CATCCTCCTGGCAGTTTTGC	CCCCTGAAGTTCTAGTGCTG G
	G7-2	Os02g55180	AK120097.1	AGGATGGCACTCAGCTGTTC	ACATGAGCTTGGTAGCTCGG
	G7-3	Os05g43480	AK120186.1	ACCAGGTATGCACCTCAAGC	TTCCAGAGTCACGGCCATTC
	G7-4	Os06g08530	AK102933	AAACAATGGCCTGGCTTCCA	TGGTTGCAATTCCTCTCTGTG A
	G7-5	Os06g44380	AK067618.1	GCGTGCGGTATTGTTCTCAA	TTCCTCGCCTCCACCTGATA
	G7-6	Os08g37350	AK099704.1	GGACCATATTCGCCAGCAGA	ATGTGACCCTGTGTCGGAAC
	G7-7	Os09g28940	AP005676.3	CCAACCAAGGTAAACACCAC G	CCCAATCGTCCTACGCTGTT
Group8	G8	Os02g46650	AK069300.1	TGAAAATGAGCAGCCCCAGT	CACATGCATTGGAGCTAGGC
Group9	G9-1	Os04g55360	AK100969.1	CGAAGCTCAGGTGGCACTAA	AACCGCTCAGCCCTTGTAAA
	G9-2	Os09g24250	AK070971.1	TTGATGCTTGGCCTTGACCT	ATACGAGCAATGGAGGGTCG
Group10	G10	Os07g46660	AK060418	TTTGGGAGCTTCACAGAGGC	TGGATCTCAGAGAGTTCCCGT
Group11	G11	Os03g09260	AK110648.1	TGTGCAGCATTTGTTCCGTG	GCTTCGGAATCCCTTCCACA
Group12	G12	Os04g34984	AK070730.1	GGACGCTCATTTTACGTGGG	AATGCCCACCTCCACATACG
Group13	G13	Os11g34270	AK065551.1	TCCAGGAGTGCTCTTTTACGG CGAA	CTCCAACTTTGAACAGCACTG GCCC
Group14	G14-1	Os09g37580	AP005862.2	TCCGATACATCTCGGGGTGA	TGATCAGTTACGGCAGGTGG
	G14-2	Os03g06950	AK062304.1	TGGGCTACACACCTGAGGAT	TCTTGATCTCATTCGGGCGG
	G14-3	Os11g34690	AK287786.1	CAAACCATGTGACCCGTGTG	GAGTGCAGCCTCCGAAAGAT
UCH	OsUCH1	Os02g57630	AK058380.1	GGGCAGTGTTCTGGTGGGCC T	CTCCAGCTCCTTCAGCTCAGC AGT
	OsUCH2	Os02g08370	AK067359.1	GCAAGGCCGGAAGTCCCCG	CGCCACCTGGGCATTTCCCC
	OsUCH3	Os02g43760	AK066320.1	TCTCGAGGCCAACCCCGACG	TCGAGGCCGTAGACGTCGCA
	OsUCH4	Os04g46190	AK059677.1	CCTCCACGGTCGAGAGCAAG AAGC	AAACGAAGCACGCTGGGCAG G
	OsUCH5	Os04g57190	AK103595.1	TGGCATGACTTCATACGAGC GTGCT	CCGGCACTGGCAGCCGATAA G

Primers for *in situ* hybridization

gene	Primer name	Primers sequence（5’-3’）
Os04g46190	Os04g46190insituF	ATTTAGGTGACACTATAGAATACGTGCTCGCCGTCCTCTTCC
	Os04g46190insituR	AATTAATACGACTCACTATAGGGCAGTGTCGCCAGCCGAAGCA
Os02g43760	Os02g43760.1insituF	ATTTAGGTGACACTATAGAATACATGTGGGGGCTGGGAGTCG
	Os02g43760.1insituR	AATTAATACGACTCACTATAGGGTCAACCAGCTTGAGTTTGGATG CAG
08g41620	08g41620insituSP6	ATTTAGGTGACACTATAGAATATGGGGTGGTAATGGACAACG
	08g41620insituasT7	AATTAATACGACTCACTATAGGGGTCCTAATATCCCTGCCCGC
08g41630	08g41630insituSP6	ATTTAGGTGACACTATAGAATACAGTTGAGTTTCTGCACGGC
	08g41630insituasT7	AATTAATACGACTCACTATAGGGATGCATGAACACCTGCCTCA
07g06610	07g06610insituSP6	ATTTAGGTGACACTATAGAATAGGGTGAATTCCAGCAGACGA
	07g06610insituasT7	AATTAATACGACTCACTATAGGGCTTTTTGCAGCATGGGCAGT
01g48600	01g48600insituSP6	ATTTAGGTGACACTATAGAATACTGCAAAAAGCATCAACAAGCG
	01g48600insituasT7	AATTAATACGACTCACTATAGGGGGTCTCACGCACTCATCATCA
02g14730	02g14730insituSP6	ATTTAGGTGACACTATAGAATAGAAGTACGGGGCATCTCAC
	02g14730insituasT7	AATTAATACGACTCACTATAGGGAAACAGGGCCACCACTTTCA
